# Practical and Sustainable Synthesis of Optically Pure Levocabastine, a H_1_ Receptor Antagonist

**DOI:** 10.3390/molecules22111971

**Published:** 2017-11-15

**Authors:** Sung Kwon Kang, Dong Hyuk Nam, Jaeseung Ahn, Jaemin Lee, Jaehoon Sim, Jeeyeon Lee, Young-Ger Suh

**Affiliations:** 1 College of Pharmacy, Seoul National University, 1 Gwanak-ro, Gwanak-gu, Seoul 08826, Korea; skkang@ckdpharm.com (S.K.K.); jaehoonsim87@gmail.com (J.S.); jyleeut@snu.ac.kr (J.L.); 2 Department of Synthetic Chemistry, Chong Kun Dang Research Institute, 315-20, Dongbaekjukjeon-daero, Giheung-gu, Yongin-si 16995, Gyeonggi-do, Korea; namdh@ckdpharm.com (D.H.N.); jaeseung@ckdpharm.com (J.A.); jmlee88@ckdpharm.com (J.L.); 3 College of Pharmacy, Cha University, 120 Haeryong-ro, Pocheon 11160, Gyeonggi-do, Korea

**Keywords:** levocabastine, anti-histamine, H_1_ receptor antagonist

## Abstract

A practical and sustainable method for the synthesis of levocabastine hydrochloride (**1**), a H_1_ receptor antagonist for the treatment of allergic conjunctivitis, that can be applied to the industrial production of the compound has been developed. Substantial improvements over the previously reported procedure are achieved via efficient preparation of an optically active key intermediate (**5**) without chiral resolution and with a more effective detosylation, which complements the previous procedure. Notably, our process requires no chromatographic purification and provides levocabastine hydrochloride in greater than 99.5% purity in a 14.2% overall yield.

## 1. Introduction

Histamine is a low-molecular-weight amine derived from the decarboxylation of histidine. It is an important mediator of local immune response as well as many biological processes related to inflammation, gastric acid secretion, and neuromodulation. Because of its potent and diverse biological activities, the systemic level of histamine must be carefully regulated during its synthesis, transport, storage, release, and degradation [1,2,3].

**Figure 1:**
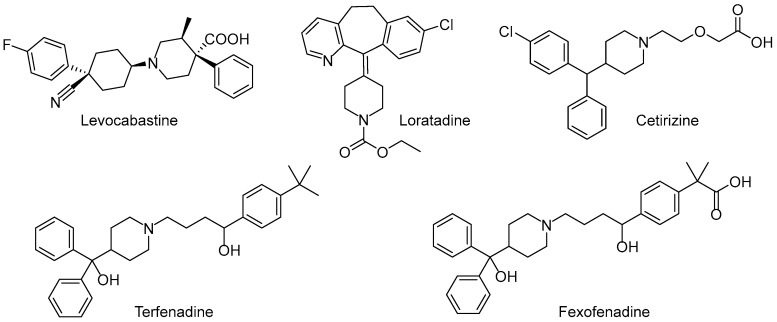
Representative H_1_ receptor antagonists.

The H_1_R, H_2_R, H_3_R, and H_4_R histamine receptors are G-protein-coupled transmembrane receptors that transduce extracellular signals. They play an important role not only in immune modulation but also in acute and chronic allergic inflammation [4]. Anti-histamines, acting as antagonists to the histamine receptors, primarily block the acute allergic response. However, the expression, signal transduction, and function of anti-histamines differ because of an affinity for each of the various histamine receptors responsible for that symptom [5,6,7,8,9,10,11,12]. In particular, the histamine H_1_ receptor has long been thought to mediate inflammatory responses by the liberation of histamine, and a number of H_1_ receptor antagonists have been used to treat allergies for many years (Figure 1).

**Scheme 1:**
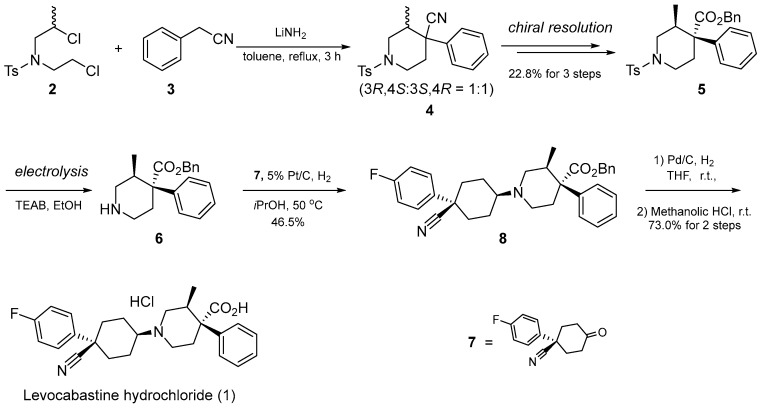
The reported synthesis of levocabastine hydrochloride.

Levocabastine hydrochloride (**1**), a selective second-generation H_1_ receptor antagonist, was discovered in 1979 by Janssen. Since then, it has been used in clinical formulations such as eye drops and nasal sprays for the treatment of allergic conjunctivitis and rhinitis [13,14,15]. Despite its clinical importance, only two synthetic methods for the preparation of levocabastine have been reported [16,17]. These processes involve the preparation of key intermediate **6** by chiral resolution, the deprotection of intermediate **5** by electrolysis, and the coupling of piperidine **6** and ketone **7** by reductive amination (Scheme 1). However, a low yield (22.8%) in the chiral resolution, the requirement of special equipment for electrolysis, and the expensive platinum catalyst for the reductive amination remain as drawbacks when adapting this process to industrial production. Thus, there has been an increasing need to develop a substantially improved method for the synthesis of levocabastine.

**Scheme 2:**
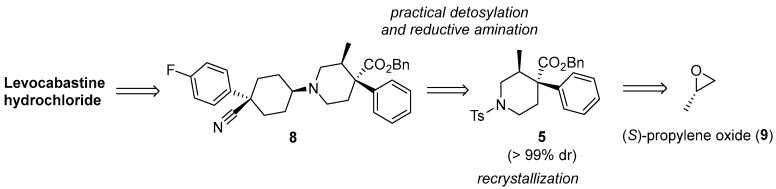
Strategy for the practically improved synthesis of levocabastine hydrochloride.

Recently, we carefully developed a practical and efficient method for the synthesis of levocabastine hydrochloride. Considering the previous elegant synthesis of levocabastine hydrochloride, we retained the concept of the previous synthetic strategy, and we focused on developing a sustainable synthetic procedure for key intermediate **5** and producing the final levocabastine with a high optical purity. Thus, the key part of our strategy involves the efficient preparation of optically pure intermediate **5** in a high yield from commercially available epoxide **9**, facile detosylation in place of electrolysis, and a high-yielding reductive coupling of amine **6** with ketone **7** to afford optically pure intermediate **8**, as outlined in Scheme 2.

## 2. Results and Discussion

### 2.1. Synthesis of Optically Pure Intermediate ***5***

**Scheme 3:**
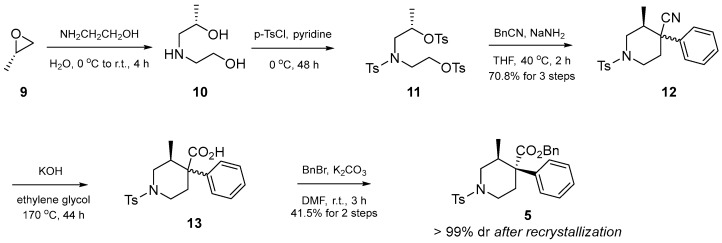
Synthesis of key intermediate **5** in an optically active form.

We avoided chiral resolution, which inherently lowers the reaction yield, in our preparation of optically pure intermediate **5** by utilizing optically active epoxide **9**, as outlined in Scheme 3.

Optically pure diol **10**, prepared by *N*-alkylation of commercially available (*S*)-propylene oxide **9** with ethanolamine, was subjected to global tosylation to provide tosylate **11**. Facile cyclization of **11** with benzyl cyanide afforded cyanide **12** as a 1:1 diastereomeric mixture. The hydrolysis of cyanide **12** with potassium hydroxide produced acid **13** as a 1:1 diastereomeric mixture. Finally, the esterification of acid **13**, followed by recrystallization, afforded key intermediate **5** with a diastereomeric purity of more than 99% dr. At this stage, we attempted the facile separation of the desired intermediate **5** by simple recrystallization of the diastereomeric mixture (instead of a delicate chiral resolution) on the basis of the distinct physical properties of the diastereomers, particularly solubility. We thoroughly explored the recrystallization of a number of intermediates (**12**, **13** and **5**) and the results are summarized in Table 1.

Recrystallization of the diastereomeric mixture of cyanide **12** only slightly improved the diastereomeric ratio (less than 53% dr) regardless of the temperature, time, or solvent (entries 1–4). In the case of acid **13**, the diastereomeric ratio was greatly improved to more than 95% dr (entries 5 and 6); however, the low yield limits the utility of this procedure. Fortunately, recrystallization of ester **5** provided a good diastereomeric ratio (over 98% dr) and a good yield (41.5%; entries 7 and 8). Accordingly, the overall yield for the preparation of ester intermediate **5** was improved by approximately 24.8% (from 4.6% to 29.4%).

### 2.2. Deprotection of Tosylate ***5***

Although intermediate **6** was obtained by electrolysis of ester **5** in a good yield according to the procedure described in a previous report [18], electrolysis requires special equipment. Thus, we looked for a convenient and economical process for detosylation under the conditions shown in Table 2 [19,20,21,22,23,24,25]. The treatment of intermediate **5** with tetra *n*-butylammonium fluoride (TBAF), thiophenol, and trimethylsilane did not provide the desired product (entries 1–4). Detosylation with low-valent titanium prepared from Ti(OiPr)_4_/Mg powder was also not successful (entry 5). Interestingly, the reaction of ester **5** with potassium diphenylphosphide (KPPh_2_) [26] provided the desired product **6** in a 68% yield (entry 6). We further optimized the detosylation conditions (entries 7–9) on the basis of entry 6. Finally, the reaction of ester **5** with potassium diphenylphosphide in tetrahydrofuran (THF) at −40 °C for 3 h afforded free amine **6** in the best yield (69.5%), although the yield was still not ideal. 

### 2.3. Completion of the Levocabastine Hydrochloride Synthesis

**Scheme 4:**
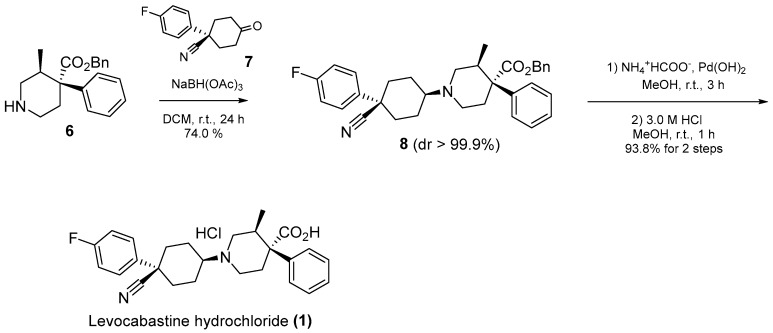
Completion of the synthesis of levocabastine hydrochloride (**1**).

The coupling of piperidine **6** with cyclohexanone **7** is an essential step for the completion of the levocabastine synthesis. In the previous synthesis, the use of an expensive platinum catalyst for reductive amination was a weak point in terms of commercial production of this compound. Thus, we focused on the development of an economical process for reductive amination using an inexpensive reducing agent. After numerous attempts, we successfully achieved the coupling of piperidine **6** and cyclohexanone **7** in the presence of NaBH(OAc)_3_. It is worth nothing that the use of NaBH(OAc)_3_ as a reducing agent without a catalyst provided amine **8** as a diastereomeric mixture (83:17) in a 95.5% yield. After recrystallization, amine **8** was obtained in a 74% yield with a high diastereomeric purity (greater than 99.9%). Having accomplished the synthesis of tertiary amine **8**, we completed the synthesis of levocabastine. The hydrogenolysis of benzyl ester **8** using ammonium formate in the presence of Pd(OH)_2_ followed by salt formation with 3.0 M HCl provided levocabastine hydrochloride **1** with an optical purity of greater than 99.9%, as outlined in Scheme 4.

## 3. Materials and Methods

### 3.1. General Information

^1^H-NMR spectra and ^13^C-NMR spectra were recorded using a Bruker DPX 400 (Bruker Biospin Gmbh, Rheinstetten, Germany) spectrometer. All purity values were obtained by HPLC analysis using HPLC 1200 Series from Agilent Technologies (Santa Clara, CA, USA). All NMR spectra were measured using 400 UltraShield NMR (Bruker Biospin Gmbh, Rheinstetten, Germany). Chemical shifts were expressed in parts per million (ppm, δ) and referenced to D_2_O (4.79 ppm for ^1^H), CDCl_3_ (7.26 ppm for ^1^H and 77.0 ppm for ^13^C), and CD_3_OD (4.87 ppm for ^1^H and 49.2 ppm for ^13^C). ^1^H-NMR data were reported in the order of chemical shift, multiplicity (s, singlet; d, doublet; q, quartet; m, multiplet; dd, doublet of doublets; td, triplet of doublets), the number of protons, and coupling constant in hertz (Hz). High-resolution mass spectra were obtained with a Synapt G2 instrument (Waters Corporation, Milford, MA, USA).

HPLC analysis (compounds **11** and **12**): YMC-Pack ODS AQ; 4.6 mm × 250 mm (3 μm); *λ* = 220 nm; flow rate: 0.5 mL/min; column temperature: 37 °C; mobile phase: (30:70) H_2_O/MeOH.

HPLC analysis (compounds **13** and **5**): YMC-Pack ODS AQ; 4.6 mm × 250 mm (3 μm); *λ* = 220 nm; flow rate: 0.5 mL/min; column temperature: 37 °C; mobile phase: (20:80) buffer: MeOH/buffer: 0.024 M Bu_4_NHSO_4_ solution.

HPLC analysis (compounds **6**, **8** and **1**): Acquity UPLC BEH Phenyl; 2.1 mm × 100 mm (1.7 μm); *λ* = 210 nm; flow rate: 0.5 mL/min; column temperature: 60 °C; mobile phase: (20/80) buffer: acetonitrile/buffer: 0.05 M Bu_4_NHSO_4_ solution.

### 3.2. Experimental Part

*(S)-1-[(2-Hydroxyethyl)amino]propan-2-ol* (**10**). To a stirred solution of ethanolamine (800.0 g, 13.10 mol) in purified water (1640 mL), (*S*)-propylene oxide (115 mL, 1.64 mol) was slowly added at 0 °C. The mixture was stirred for 1 h at the same temperature and for 4 h at room temperature. The reaction mixture was concentrated under reduced pressure at 90 °C. Diol **10** was obtained as a yellow oil (207.0 g). This product was used in the next step without further purification; ^1^H-NMR (400 MHz, D_2_O): δ 1.15 (d, 3H, *J* = 6.4 Hz), 2.57–2.59 (m, 2H), 2.70–2.73 (m, 2H), 3.66 (td, 2H, *J* = 5.7 Hz, 2.1 Hz), 3.91 (dd, 1H, *J* = 6.4 Hz, 5.9 Hz). ^13^C-NMR (100 MHz, CDCl_3_): δ 64.8, 59.6, 56.1, 50.9, 21.0. HRMS: Calcd. for C_5_H_13_NO_2_ [M + H]^+^ 120.1025; found 120.1025.

*(S)-1-({4-Methyl-N-[2-(tosyloxy)ethyl]phenyl}sulfonamido)propan-2-yl-4-methyl benzenesulfonate* (**11**). To a stirred solution of *p*-toluenesulfonylchlrode (260.0 g, 1.36 mol) in pyridine (75 mL), diol **10** (50.0 g) in pyridine (200 mL) was slowly added at 0 °C. The mixture was stirred for 48 h at the same temperature and quenched with H_2_O (700 mL) and dichloromethane (700 mL) at room temperature. The reaction slurry was washed with 4.0 M HCl solution (700 mL) and 10% NaCl solution (900 mL) and was concentrated in vacuo to afford tosylate **11** as a yellow oil (233.0 g). This product was used in the next step without further purification; ^1^H-NMR (400 MHz, CDCl_3_): δ 1.21 (d, 3H, *J* = 6.4 Hz), 2.43 (s, 3H), 2.45 (s, 3H), 2.46 (s, 3H), 3.15–3.28 (m, 3H), 3.33–3.39 (m, 1H), 4.06–4.13 (m, 2H), 4.77 (q, 1H, *J* = 6.2 Hz), 7.26–7.36 (m, 6H), 7.61 (d, 2H, *J* = 8.3 Hz), 7.73–7.78 (m, 4H). ^13^C-NMR (100 MHz, CDCl_3_): δ 145.3, 145.1, 144.3, 135.0, 133.7, 132.5, 130.1, 128.1, 128.0, 127.5, 77.8, 68.2, 54.4, 49.0, 25.5, 21.8, 21.7, 18.3. HRMS: Calcd. for C_26_H_31_NO_8_S_3_ [M + Na]^+^ 604.1110; found 604.1111.

*(3S)-3-Methyl-4-phenyl-1-tosylpiperidine-4-carbonitrile* (**12**). To a suspension of sodium amide (48.5 g, 1.24 mol) in THF (675 mL), a solution of benzyl cyanide (115 mL, 1.64 mol) in THF (460 mL) was slowly added at 0 °C. The mixture was stirred for 1 h at room temperature, and a solution of tosylate **11** (233.0 g) in THF was added dropwise for 1 h at 0 °C. The reaction mixture was stirred for 2 h at 40 °C, cooled to 0 °C, and 15% NH_4_Cl solution (140 mL) was added. The reaction slurry was distilled until the THF was completely removed. To the concentrated mixture, purified water (1000 mL) and dichloromethane (1000 mL) were added, and the reaction slurry was stirred for 30 min. The organic layer was washed with 10% NaCl solution (1000 mL) and concentrated under reduced pressure. The residue was dissolved with MeOH (230 mL). The resulting solution was stirred for 1 h at 75 °C, for 30 min at room temperature, and for 1 h at 0 °C. The precipitate was filtered and then dried to afford cyanide **12** as a white solid (80.3 g, 70.8% for three steps); ^1^H-NMR (400 MHz, CDCl_3_): δ 0.81–0.85 (m, 3H), 2.09–2.13 (m, 1H), 2.26–2.44 (m, 2H), 2.48–2.49 (m, 3H), 2.58–3.04 (m, 2H), 3.74–3.91 (m, 1H), 3.96–4.09 (m, 1H), 7.28–7.46 (m, 7H), 7.68–7.72 (m, 2H). ^13^C-NMR (100 MHz, CDCl_3_): δ 144.2, 144.1, 137.8, 137.6, 133.2, 133.0, 130.1, 129.3, 128.6, 127.7, 127.6, 126.3, 126.0, 122.6, 119.2, 50.0, 49.7, 49.5, 44.2, 44.0, 43.8, 39.1, 38.3, 37.8, 26.6, 21.7, 14.2, 12.4. HRMS: Calcd. for C_20_H_22_N_2_O_2_S [M + H]^+^ 355.1480; found 355.1481; dr = 53.1:46.9.

*(3S)-3-Methyl-4-phenyl-1-tosylpiperidine-4-carboxylic acid* (**13**). To a stirred solution of cyanide **12** (80.3 g, 0.23 mol) in ethylene glycol (400 mL), potassium hydroxide (89.0 g, 1.59 mol) was added. The reaction mixture was stirred for 44 h at 170 °C, cooled to room temperature, and dichloromethane (562 mL) was added. The reaction slurry was slowly quenched with 2.0 M HCl solution (900 mL) at 0 °C. The organic layer was washed with 10% NaCl solution (800 mL) and concentrated in vacuo to afford acid **13** as a yellow oil (92.3 g). This product was used in the next step without further purification; ^1^H-NMR (400 MHz, CDCl_3_): δ 0.78–1.14 (m, 3H), 2.28–2.39 (m, 2H), 2.41–2.45 (m, 3H), 2.53–2.90 (m, 2H), 3.00–3.18 (m, 2H), 3.62–3.95 (m, 1H), 7.25–7.38 (m, 8H), 7.58–7.66 (m, 2H). ^13^C-NMR (100 MHz, CDCl_3_): δ 179.3, 179.0, 143.6, 140.3, 139.0, 133.8, 129.9, 129.8, 129.0, 128.9, 127.7, 127.6, 127.5, 127.0, 126.1, 52.8, 52.1, 50.1, 48.5, 44.4, 43.1, 34.4, 25.9, 21.7, 21.6, 14.8, 13.5. HRMS: Calcd. for C_20_H_23_NO_4_S [M + H]^+^ 374.1426; found 374.1423.

*(3S,4R)-Benzyl-3-methyl-4-phenyl-1-tosylpiperidine-4-carboxylate* (**5**). To a stirred solution of acid **13** (92.3 g) in DMF (320 mL), potassium carbonate (37.6 g, 0.27 mol) and benzyl bromide (32.4 g, 0.27 mol) were added. The reaction mixture was stirred for 3 h at room temperature, cooled to 0 °C, and quenched with 10% NH_4_Cl solution (600 mL) and ethyl acetate (500 mL). The organic layer was washed with 10% NaCl solution (500 mL) and concentrated under reduced pressure. The residue was clearly dissolved with MeOH (60 mL). To the resulting solution, isopropyl ether (360 mL) was added, and the solution was stirred at room temperature for 15 h and at 0 °C for an additional 1 h. The precipitate was filtered and then dried to afford ester **5** as a white solid (42.9 g, 41.5% for two steps); ^1^H-NMR (400 MHz, CDCl_3_): δ 0.79 (d, 3H, *J* = 7.0 HZ), 2.19–2.29 (m, 2H), 2.46 (s, 3H), 2.56–2.62 (m, 2H), 2.92–2.95 (m, 1H), 3.57–3.61 (m, 1H), 3.87–3.90 (m, 1H), 4.93 (dd, 2H, *J* = 21.2 Hz, 12.3 Hz), 6.96–6.98 (m, 2H), 7.17–7.21 (m, 2H), 7.23–7.33 (m, 8H), 7.57–7.60 (m, 2H). ^13^C-NMR (100 MHz, CDCl_3_): δ 173.8, 143.4, 140.6, 135.5, 133.4, 129.8, 128.8, 128.5, 128.2, 127.9, 127.6, 127.5, 126.0, 66.8, 52.4, 50.2, 44.5, 34.5, 25.9, 21.7, 13.5. HRMS: Calcd. for C_27_H_29_NO_4_S [M + H]^+^ 464.1896; found 464.1894; dr = 99.4:0.6.

Convenient detosylation of intermediate **5** (Table 2).

*(3S,4R)-Benzyl-3-methyl-4-phenylpiperidine-4-carboxylate* (**6**, entry 1 in Table 2). To a stirred solution of ester **5** (46 mg, 0.10 mmol) in THF (10 mL), TBAF in THF (1.00 mmol) was added at room temperature. The reaction mixture was stirred for 5 h under refluxing conditions. No reaction (by TLC) was observed.

*(3S,4R)-Benzyl-3-methyl-4-phenylpiperidine-4-carboxylate* (**6**, entry 2 in Table 2). To a stirred solution of ester **5** (46 mg, 0.10 mmol) in THF (10 mL) and acetonitrile (10 mL), potassium carbonate (28 mg, 0.20 mmol) and thiophenol (14 mg, 0.13 mmol) were added at room temperature. The reaction mixture was stirred for 21 h under refluxing conditions. No reaction (by TLC) was observed.

*(3S,4R)-Benzyl-3-methyl-4-phenylpiperidine-4-carboxylate* (**6**, entry 3 in Table 2). To a suspension of sodium iodide (23 mg, 0.15 mmol) in ACN (10 mL), trimethylsilyl chloride (16 mg, 0.15 mmol) was slowly added at 0 °C. To the mixture, ester **5** (46 mg, 0.10 mmol) was added. The reaction mixture was stirred for 17 h under refluxing conditions. No reaction (by TLC) was observed.

*(3S,4R)-Benzyl-3-methyl-4-phenylpiperidine-4-carboxylate* (**6**, entry 4 in Table 2). To a reaction flask, ester **5** (49 mg, 0.11 mmol) and iodotrimethylsilane (1 mL, 0.75 mmol) were added. The reaction mixture was stirred for 1 h at 80 °C. No reaction (by TLC) was observed.

*(3S,4R)-Benzyl-3-methyl-4-phenylpiperidine-4-carboxylate* (**6**, entry 5 in Table 2). To a stirred solution of ester **5** (100 mg, 0.22 mmol) in THF (10 mL), Mg (26 mg, 1.08 mmol) was added at room temperature. The mixture was stirred for 30 min at the same temperature and titanium isopropoxide (61 mg, 0.22 mmol) and trimethylsilyl chloride (35 mg, 0.32 mmol) were added. The reaction mixture was stirred 21 h at 50 °C. No reaction (by TLC) was observed.

*(3S,4R)-Benzyl-3-methyl-4-phenylpiperidine-4-carboxylate* (**6**, entry 7 in Table 2). To a stirred solution of ester **5** (50.0 g, 0.11 mol) in THF (500 mL), 0.5 M potassium diphenylphosphide solution (280 mL, 0.14 mol) was added dropwise at −40 °C. The mixture was stirred for 3 h at the same temperature. To the reaction slurry, 2.0 M HCl solution (250 mL) was added, and the mixture was stirred for 2 h at room temperature. The reaction mixture was quenched with 10% NaHCO_3_ solution (1000 mL) and ethyl acetate (800 mL). The organic layer was concentrated under reduced pressure to afford piperidine **6** as a yellow oil (67.5 g). This product was used in the next step without further purification; ^1^H-NMR (400 MHz, CD_3_OD): δ 0.75 (d, 3H, *J* = 7.4 Hz), 2.27 (td, 1H, *J* = 13.7 Hz, 4.2 Hz), 2.59–2.63 (m, 1H), 2.75 (td, 1H, *J* = 13.4 Hz, 2.8 Hz), 2.97–3.00 (m, 1H), 3.09–3.24 (m, 2H), 3.31–3.35 (m, 1H), 5.12 (dd, 2H, *J* = 12.2 Hz, 5.8 Hz), 7.13–7.15 (m, 2H), 7.24–7.37 (m, 8H). ^13^C-NMR (100 MHz, CD_3_OD): δ 174.8, 141.7, 137.1, 129.9, 129.5, 129.3, 129.2, 128.6, 126.9, 68.1, 53.1, 43.8, 34.5, 25.5, 13.4. HRMS: Calcd. for C_20_H_23_NO_2_ [M + H]^+^ 310.1807; found 310.1811.

*(3S,4R)-Benzyl-1-[(1S,4R)-4-cyano-4-(4-fluorophenyl)cyclohexyl]-3-methyl-4-phenylpiperidine-4-carboxylate* (**8**). To a suspension of piperidine **6** (67.5 g) in dichloromethane (400 mL), ketone **7** (56.2 g, 0.26 mol) and NaBH(OAc)_3_ (57.2 g, 0.27 mol) were added at room temperature. The reaction mixture was stirred for 24 h and quenched with 10% NaHCO_3_ solution (700 mL) at the same temperature. The organic layer was washed with 10% NaCl solution (600 mL), and concentrated in vacuo. The residue was clearly dissolved with methanol (500 mL) at 60 °C. The resulting solution was stirred for 2 h at room temperature. The precipitate was filtered and then dried to afford tertiary amine **8** as an off-white solid (28.4 g, 51.5% for two steps); ^1^H-NMR (400 MHz, CDCl_3_): δ 0.76 (d, 3H, *J* = 7.0 Hz), 1.76–1.91 (m, 6H), 2.19–2.24 (m, 3H), 2.32–2.38 (m, 2H), 2.57–2.60 (m, 1H), 2.65–2.71 (m, 2H), 2.94 (d, 2H, *J* = 9.2 Hz), 5.11 (dd, 2H, *J* = 24.6 Hz, 12.4 Hz), 7.08–7.12 (m, 2H), 7.17–7.20 (m, 2H), 7.24–7.39 (m, 8H), 7.46–7.49 (m, 2H). ^13^C-NMR (100 MHz, CDCl_3_): δ 174.8, 163.5, 161.1, 141.9, 136.6, 136.1, 128.6, 128.5, 128.1, 127.5, 127.4, 127.0, 126.3, 122.4, 116.0, 115.8, 66.6, 62.3, 53.2, 53.0, 48.2, 43.8, 37.2, 35.1, 27.2, 26.2, 25.0, 14.6. HRMS: Calcd. for C_33_H_35_N_2_O_2_F [M + H]^+^ 511.2761; found 511.2762. HPLC purity: 99.5%. Geometric isomer < 0.1%.

Levocabastine hydrochloride (**1**). To a stirred solution of tertiary amine **8** (51.3 g, 0.10 mol) in dichloromethane (210 mL) and MeOH (420 mL), ammonium formate (12.7 g, 0.20 mol) and palladium hydroxide (5.1 g, 10 wt % compound **8**) were added at room temperature. The reaction mixture was stirred for 3 h at the same temperature, and 7.0 M ammonia in methanol (500 mL) was slowly added. The reaction slurry was concentrated under reduced pressure. To the concentrated mixture, MeOH (255 mL) and IPE (1020 mL) were added, and the reaction slurry was stirred for 1 h at room temperature. The precipitate was filtered. The obtained solid was suspended with MeOH (208 mL). To a suspended solution, 3.0 M HCl solution in MeOH (165 mL) was slowly added, and then the reaction mixture was stirred for 30 min at room temperature. To the reaction slurry, IPE (754 mL) was added, and the mixture was stirred for 1 h at room temperature. The solid was filtered and then washed with IPE (500 mL). The residue was suspended with EtOH (132 mL) and MeOH (132 mL) for 6 h at 50 °C and cooled to room temperature. The solid was filtered, washed with ethanol (264 mL), and then dried to afford levocabastine hydrochloride **1** as a white solid (43.1 g, 93.8% for two steps); ${\left[\alpha \right]}_{D}^{20}$ = −104.02 (c = 1, MeOH). ^1^H-NMR (400 MHz, CD_3_OD): δ 0.86 (d, 3H, *J* = 7.6 Hz), 1.97–2.13 (m, 4H), 2.35–2.54 (m, 5H), 2.83 (dd, 1H, *J* = 12.6 Hz, 2.0 Hz), 3.12 (td, 1H, *J* = 13.3 Hz, 2.6 Hz), 3.21–3.23 (m, 1H), 3.41–3.45 (m, 2H), 3.70–3.74 (m, 1H), 3.82–3.85 (m, 1H), 7.16–7.21 (m, 2H), 7.31–7.35 (m, 1H), 7.38–7.42 (m, 4H), 7.58–7.62 (m, 2H). ^13^C-NMR (100 MHz, CD_3_OD): δ 176.1, 165.1, 162.7, 141.0, 137.0, 136.9, 130.0, 128.9, 128.8, 126.9, 122.7, 117.0, 116.8, 65.4, 54.9, 52.0, 50.0, 44.1, 36.5, 35.0, 25.9, 25.5, 25.4, 13.9. HRMS: Calcd. for C_26_H_29_N_2_O_2_F [M + H]^+^ 421.2291; found 421.2292. HPLC purity: 99.7%. Optical purity > 99.9%.

## 4. Conclusions

In summary, a practical and sustainable method for the synthesis of levocabastine hydrochloride with a high purity (>99.5% by HPLC) was accomplished in a 14.2% overall yield through nine steps from commercially available and optically pure epoxide **9**. The high optical purity was achieved via simple recrystallization without chiral resolution. Our synthetic procedure enabled us to replace the previous process for detosylation by electrolysis and reductive amination using an expensive metal catalyst with convenient and economical methods, respectively. Our synthetic procedure seems industrially suitable and could be widely utilized to secure pharmaceutically useful molecules.

## Figures and Tables

**Table 1:** Recrystallization of intermediates **12**, **13** and **5**.

Entry	Intermediate	Temperature	Time (h)	Solvent	Diastereomeric Ratio of 12, 13 and 5 *^a^*	Diastereomeric Ratio of 12, 13 and 5 after Recrystallization *^a^*	Yield (%) *^b^*
1	**12**	Reflux to r.t.	1	MeOH	40:60	53:47:00	70.8
2	**12**		1	MeOH/CHCl_3_	40:60	48:52:00	68.3
3	**12**		1	Acetone	40:60	50:50:00	66.5
4	**12**	R.t.	6	MeCN/MeOH	40:60	46:54:00	71.2
5	**13**	Reflux to r.t.	1	IPE	50:50:00	98:02:00	23
6	**13**	40 °C	1.5	IPE	50:50:00	95:05:00	22.9
7	5	R.t. to 0 °C	16	MeOH/IPE	52:48:00	99:01:00	41.5
8	**5**		16	MeOH/HEX/IPE	52:48:00	98:02:00	38.2

*^a^* Diastereoselectivity was determined by HPLC; *^b^* Isolated yield. Abbreviations: r.t.—room temperature, IPE—diisopropyl ether, HEX—*n*-hexane.

**Table 2:**
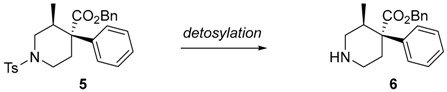
Convenient detosylation of intermediate **5**.

Entry	Reagents (eq.)	Temperature (°C)	Reaction Time (h)	Solvent	Yield (%) *^a^*
1	TBAF (10.0)	Reflux	5	THF	NR *^b^*
2	Thiophenol (1.3)/K_2_CO_3_ (2.0)		21	DMF/MeCN	NR
3	TMSCl (1.5)/NaI (1.5)		17	MeCN	NR
4	TMSI (neat)	80	1	—	NR
5	Mg (5.0)/Ti(OiPr)_4_ (1.0)	50	21	THF	NR
6	0.5 M KPPh_2_ in THF (1.3)	−78	24		68
7	0.5 M KPPh_2_ in THF (1.3)	−40	3		69.5
8		−20	3		43.7
9		0	3		30.4

*^a^* Isolated yield; *^b^* No reaction. Abbreviations: DMF—dimethylformamide, TMSCl—trimethylsilyl chloride, TMSI—trimethylsilyl iodide, HEX—*n*-hexane).

## Supplementary Materials

Supplementary data associated with this article can be found in the SI: NMR spectra and HR-MS spectra of compounds **10**, **11**, **12**, **13**, **5**, **6**, **8** and **1**; HPLC spectra of compounds **12**, **5**, **8** and **1**.
