# Supplementary material for: Practical and Sustainable Synthesis of Optically Pure Levocabastine, a H1 Receptor Antagonist
Source: Molecules. 2017 Nov 15;22(11):1971. doi: 10.3390/molecules22111971 (PMC6150193; doi:10.3390/molecules22111971)
Supplement: Supplementary file 1 [file molecules-22-01971-s001.pdf]

Supporting Information

Practical and Sustainable Synthesis of Optically Pure Levocabastine, an H_1 receptor antagonist

Sung Kwon Kang[1, 2], Dong Hyuk Nam[2], Jaeseung Ahn[2], Jaemin Lee[2], Jaehoon Sim[1], Jeeyeon Lee[1], and Young-Ger Suh[1,*]

[1] College of Pharmacy, Seoul National University, 1 Gwanak-ro, Gwanak-gu, Seoul 151-742, Korea; skkang@ckdpharm.com; jaehoonsim87@gmail.com
[2] Department of Synthetic Chemistry, Chong Kun Dang Research Institute, 315-20, Dongbaekjukjeon-daero, Giheung-gu, Yongin-si, Gyeonggi-do, Korea; namdh@ckdpharm.com; jaeseung@ckdpharm.com; jmlee88@ckdpharm.com
* Correspondence: ygsuh@snu.ac.kr; Tel.: +82-2-880-9174

Contents:

1. Compound **10** (^{1}H-NMR, ^{13}C-NMR, HR-MS) 2
2. Compound **11** (^{1}H-NMR, ^{13}C-NMR, HR-MS) 3
3. Compound **12** (^{1}H-NMR, ^{13}C-NMR, HR-MS, HPLC) 5
4. Compound **13** (^{1}H-NMR, ^{13}C-NMR, HR-MS) 8
5. Compound **5** (^{1}H-NMR, ^{13}C-NMR, HR-MS, HPLC) 9
6. Compound **6** (^{1}H-NMR, ^{13}C-NMR, HR-MS) 12
7. Compound **8** (^{1}H-NMR, ^{13}C-NMR, HR-MS, HPLC) 13
8. Compound **1** (^{1}H-NMR, ^{13}C-NMR, HR-MS, HPLC) 17

(*S*)-1-((2-hydroxyethyl)amino)propan-2-ol (10)

▼ ^{1}H-NMR (400MHz, D_2O) spectrum

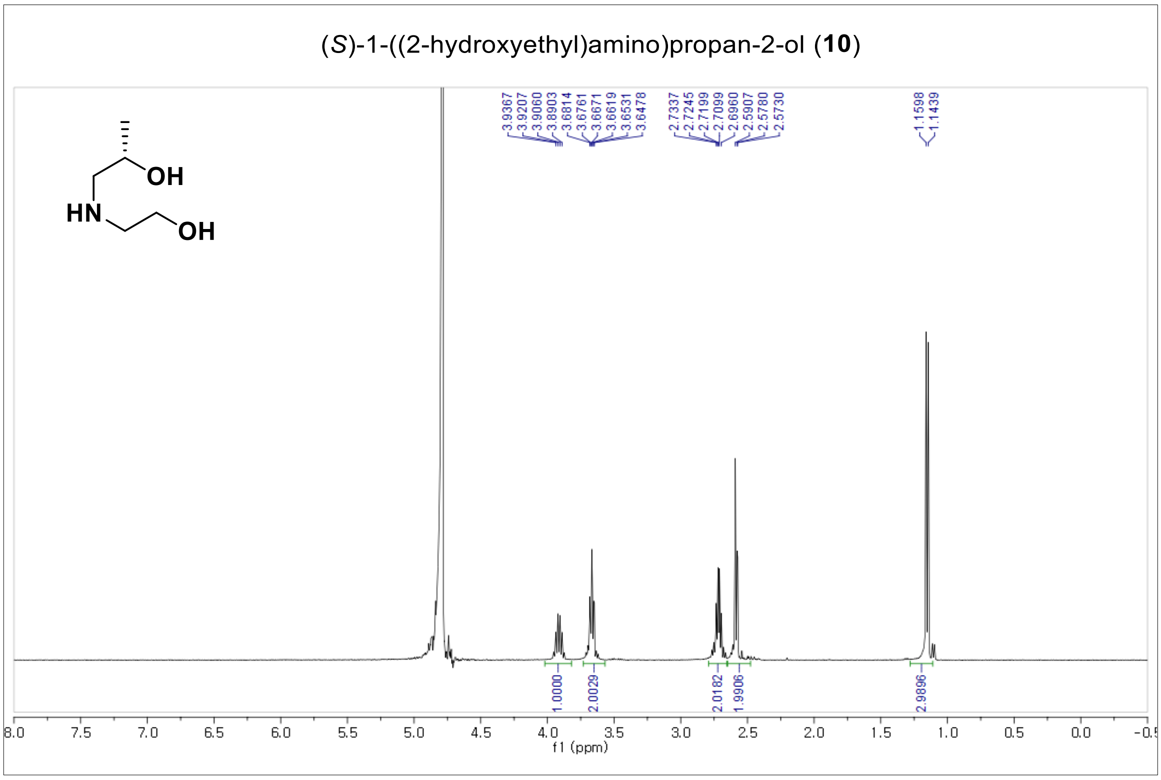

▼ ^{13}C-NMR (100MHz, $CDCl_3$) spectrum

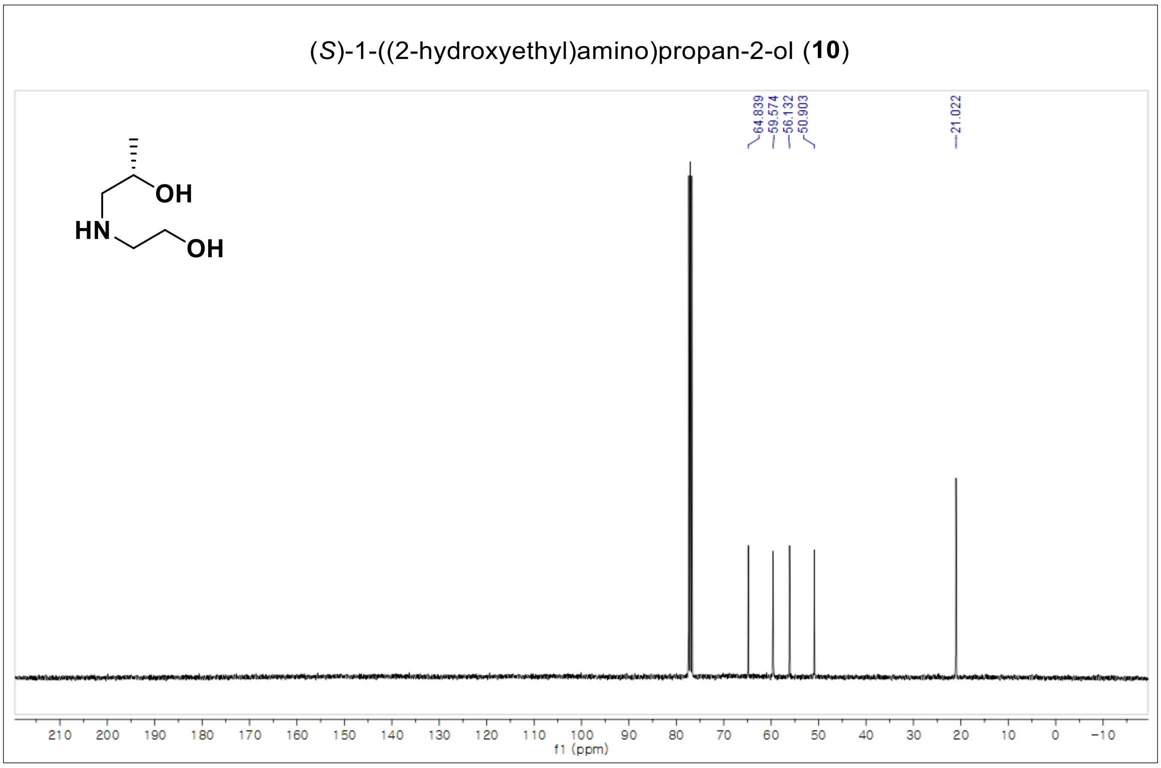

▼ High-Resolution Mass spectrum

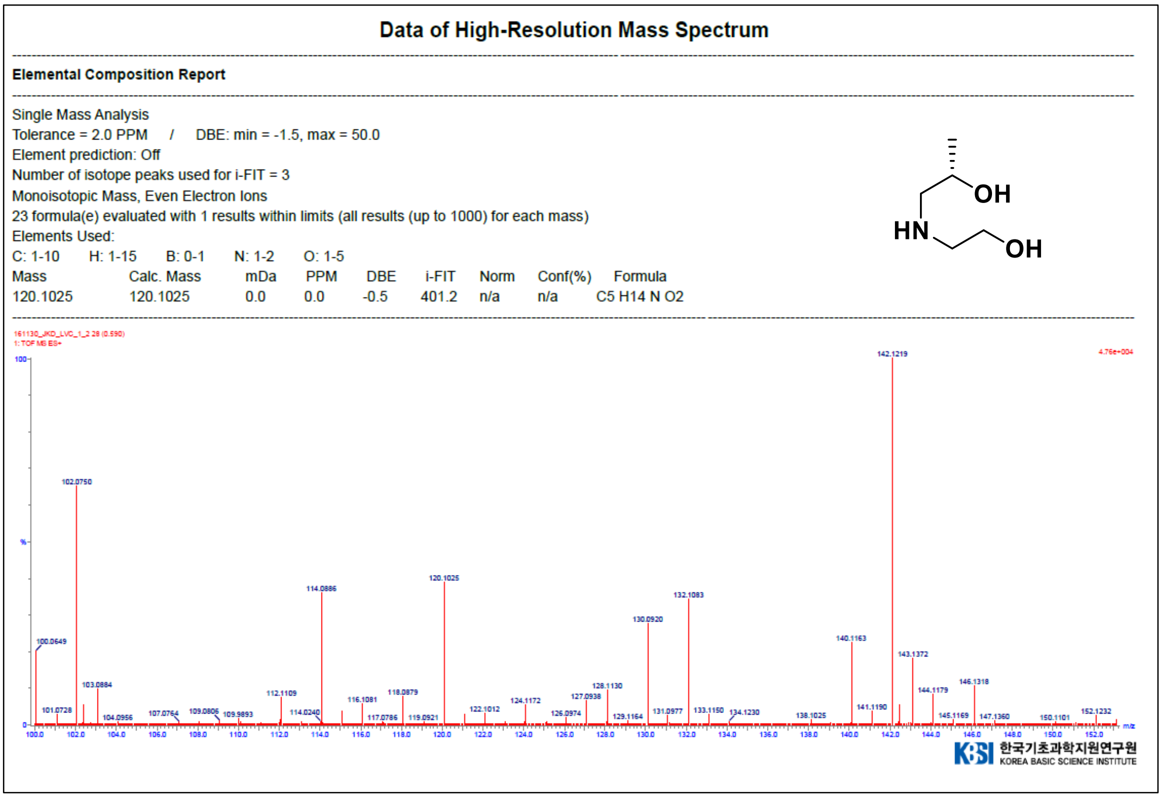

(*S*)-1-((4-methyl-*N*-(2-(tosyloxy)ethyl)phenyl)sulfonamido)propan-2-yl-4-methyl benzenesulfonate (11)

▼ ^{1}H-NMR (400MHz, $CDCl_3$) spectrum

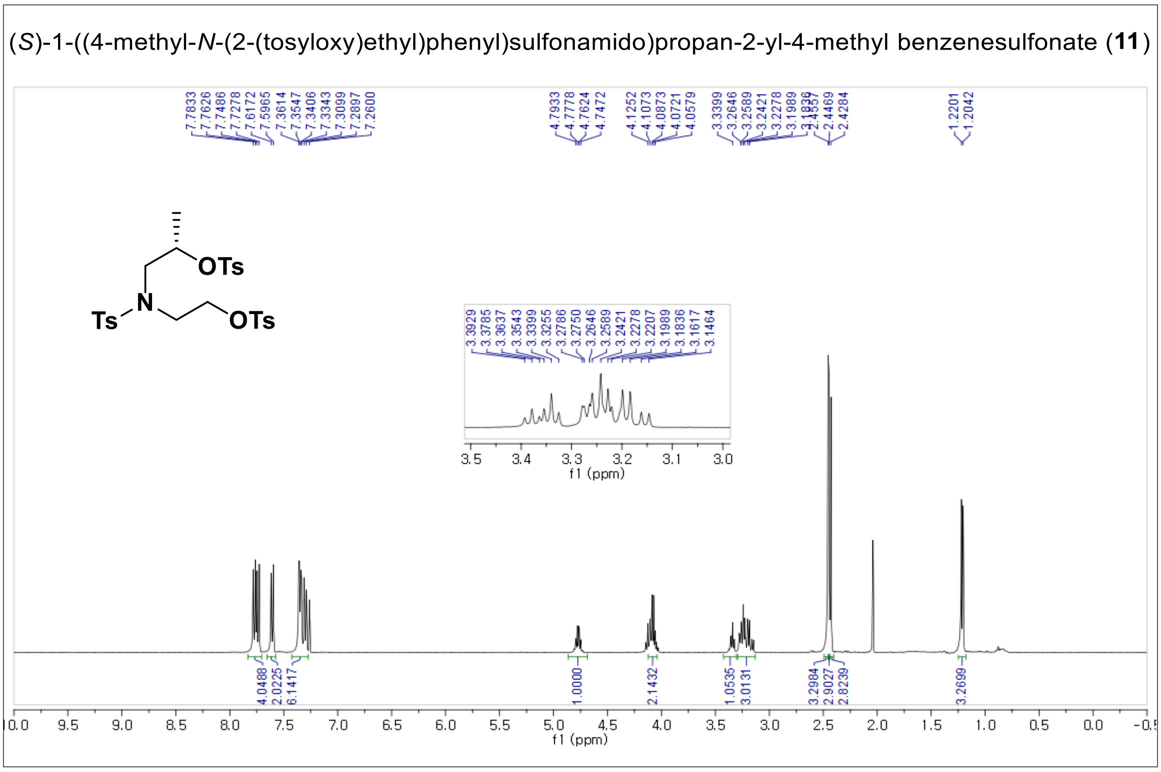

▼ ^{13}C-NMR (100MHz, $CDCl_3$) spectrum

(*S*)-1-((4-methyl-*N*-(2-(tosyloxy)ethyl)phenyl)sulfonamido)propan-2-yl-4-methyl benzenesulfonate (**11**)

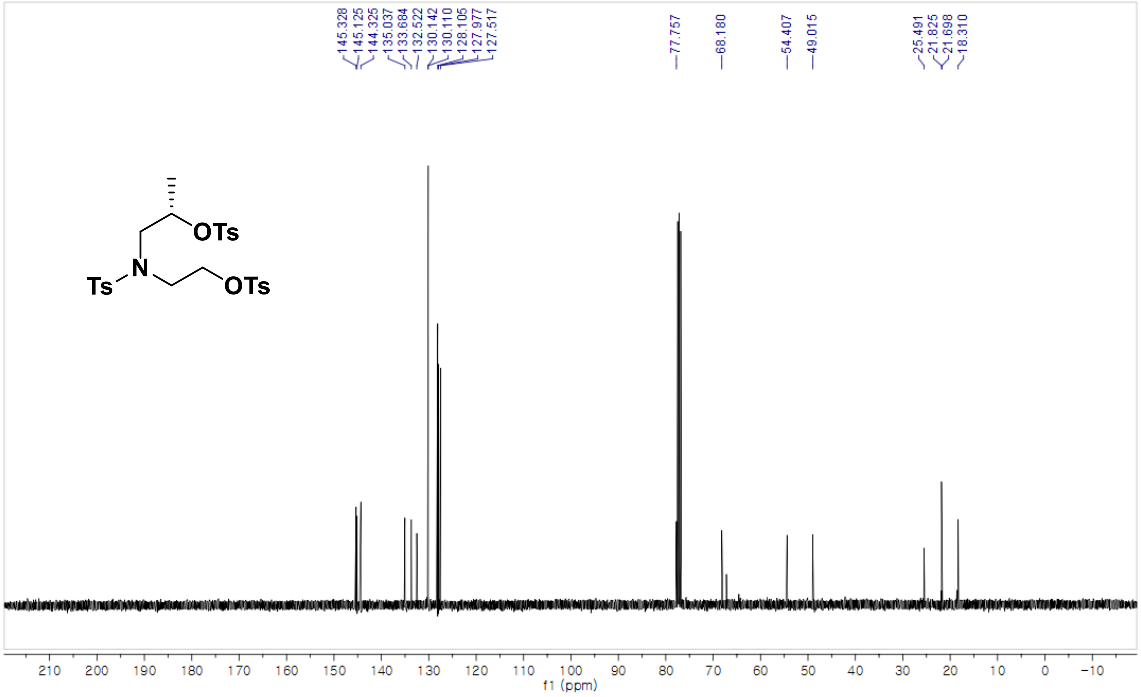

▼ High-Resolution Mass spectrum

Data of High-Resolution Mass Spectrum

Elemental Composition Report

Single Mass Analysis
Tolerance = 2.0 PPM / DBE: min = -1.5, max = 50.0
Element prediction: Off
Number of isotope peaks used for i-FIT = 3
Monoisotopic Mass, Even Electron Ions
309 formula(e) evaluated with 1 results within limits (all results (up to 1000) for each mass)
Elements Used:
C: 1-30 H: 1-35 B: 0-1 N: 1-2 O: 1-10 Na: 0-1 S: 1-3

Mass	Calc. Mass	mDa	PPM	DBE	i-FIT	Norm	Conf(%)	Formula
604.1111	604.1110	0.1	0.2	11.5	129.6	n/a	n/a	C26 H31 N O8 Na S3

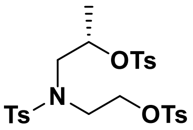

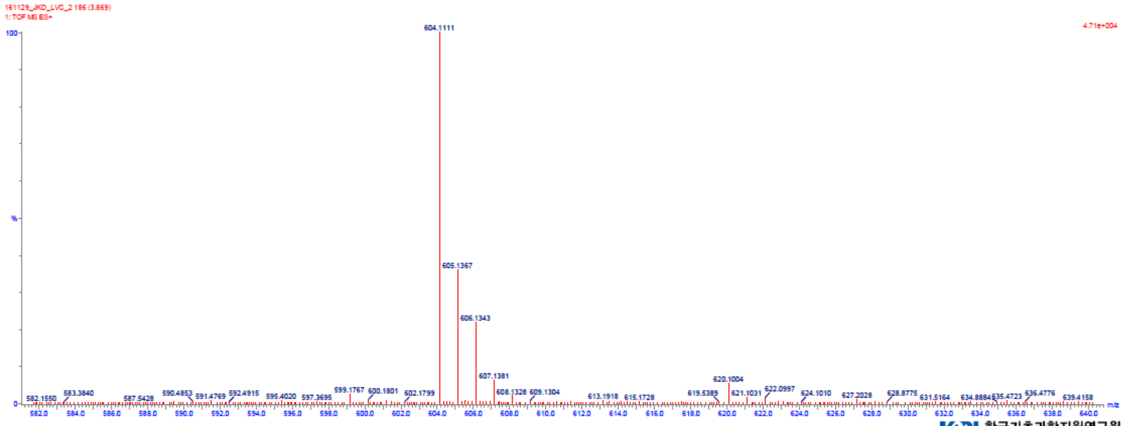

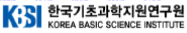

(3*S*)-3-methyl-4-phenyl-1-tosylpiperidine-4-carbonitrile (12)

▼ ^{1}H-NMR (400MHz, $CDCl_3$) spectrum

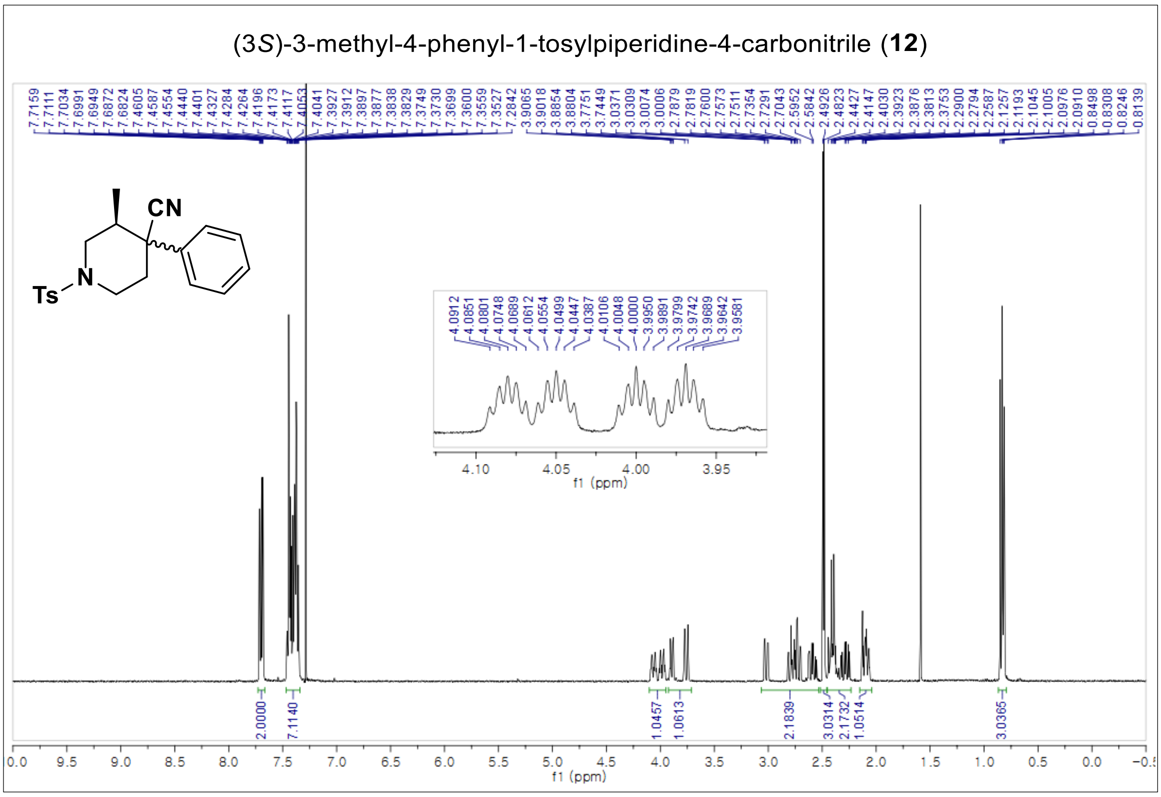

▼ ^{13}C-NMR (100MHz, $CDCl_3$) spectrum

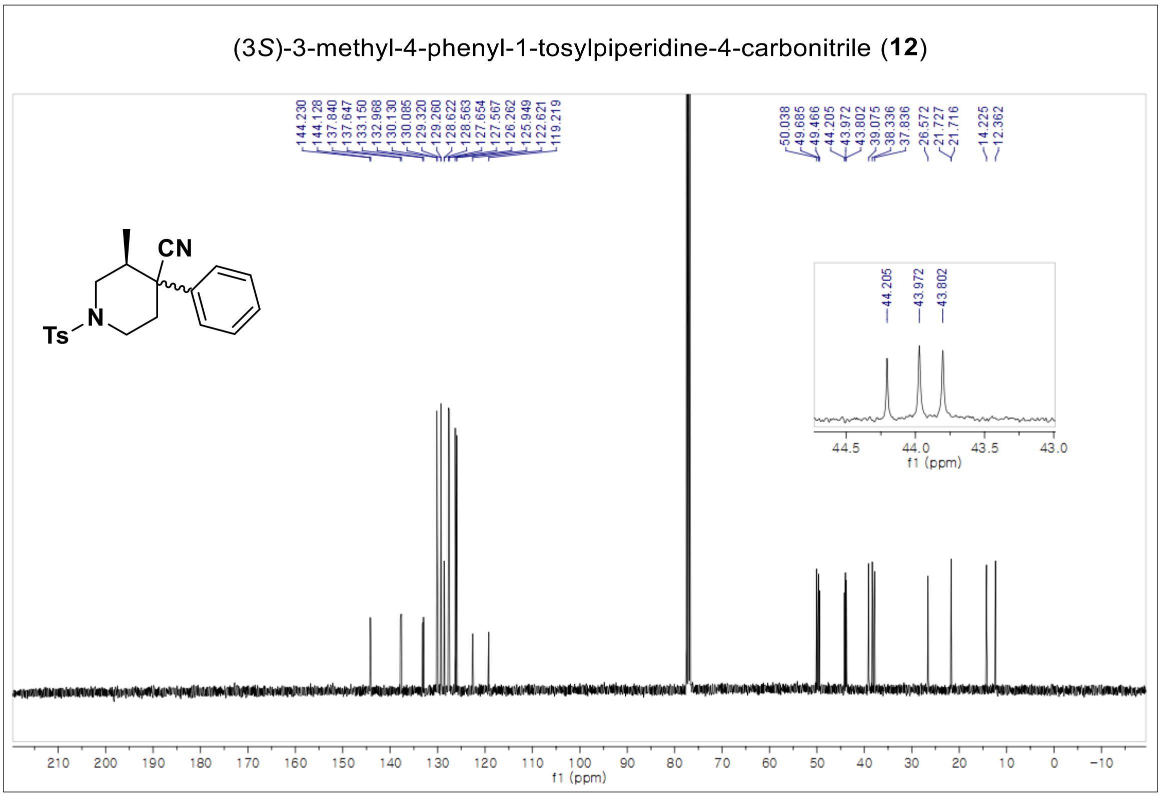

▼ High-Resolution Mass spectrum

Data of High-Resolution Mass Spectrum

Elemental Composition Report

Single Mass Analysis
Tolerance = 2.0 PPM / DBE: min = -1.5, max = 50.0
Element prediction: Off
Number of isotope peaks used for i-FIT = 3
Monoisotopic Mass, Even Electron Ions
329 formula(e) evaluated with 1 results within limits (all results (up to 1000) for each mass)
Elements Used:
C: 1-30 H: 1-35 B: 0-1 N: 1-2 O: 1-10 S: 1-3

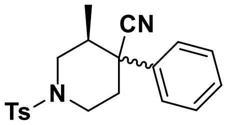

Mass	Calc. Mass	mDa	PPM	DBE	i-FIT	Norm	Conf(%)	Formula
355.1481	355.1480	0.1	0.3	10.5	187.4	n/a	n/a	C20 H23 N2 O2 S

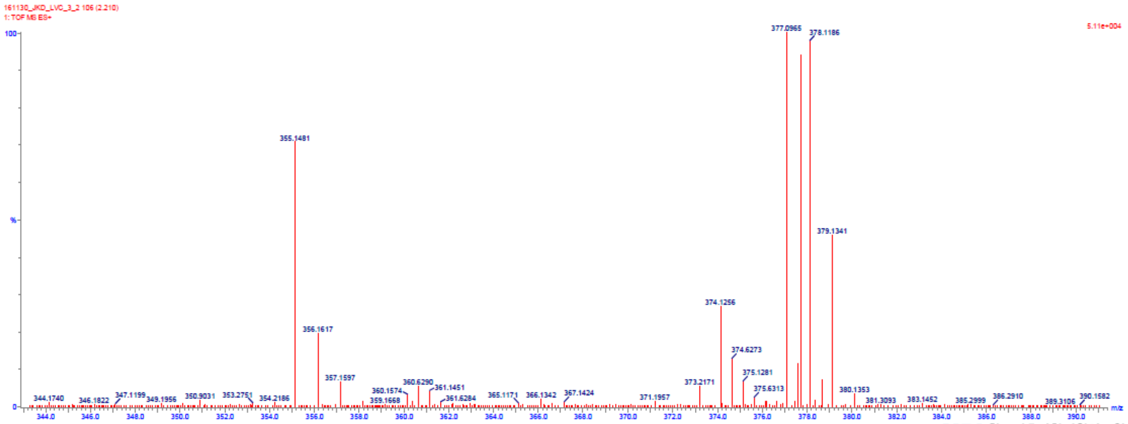

KBSI 한국기초과학지원연구원
KOREA BASIC SCIENCE INSTITUTE

▼ HPLC (dr = 53.1 : 46.9)

===

Acq. Operator : LJM Seq. Line : 11
Acq. Instrument : HPLC1200 Location : Vial 70
Injection Date : 10/7/2015 1:46:18 PM Inj : 1
Inj Volume : 10.000 µl
Acq. Method : C:\CHEM32\1\DATA\LVC-3\LVC-3 2015-10-07 19-14-56\LVC-4.M
Last changed : 10/7/2015 2:53:43 AM by KSJ
Analysis Method : C:\CHEM32\1\METHODS\DIQUAFOSOL_ASSAY.M
Last changed : 12/1/2016 10:56:53 AM by cgh
(modified after loading)
Sample Info : 18.2 mg / 20 ml

Additional Info : Peak(s) manually integrated

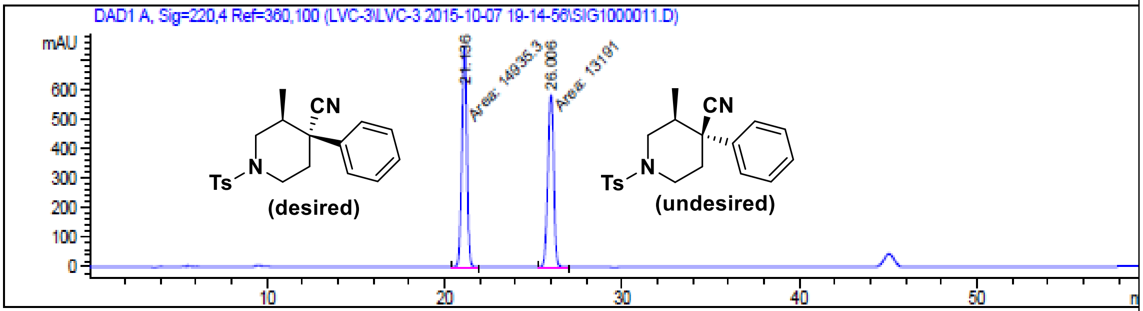

===

Area Percent Report

===

Sorted By : Signal
Multiplier: : 1.0000
Dilution: : 1.0000
Use Multiplier & Dilution Factor with ISTDs

Signal 1: DAD1 A, Sig=220,4 Ref=360,100

Peak #	RetTime [min]	Type	Width [min]	Area [mAU*s]	Height [mAU]	Area %
1	21.136	MM	0.3315	1.49353e4	750.86993	53.1007
2	26.006	MM	0.3781	1.31910e4	581.53333	46.8993

Totals : 2.81263e4 1332.40326

===

*** End of Report ***

(3*S*)-3-methyl-4-phenyl-1-tosylpiperidine-4-carboxylic acid (13)

▼ ^{1}H-NMR (400MHz, $CDCl_3$) spectrum

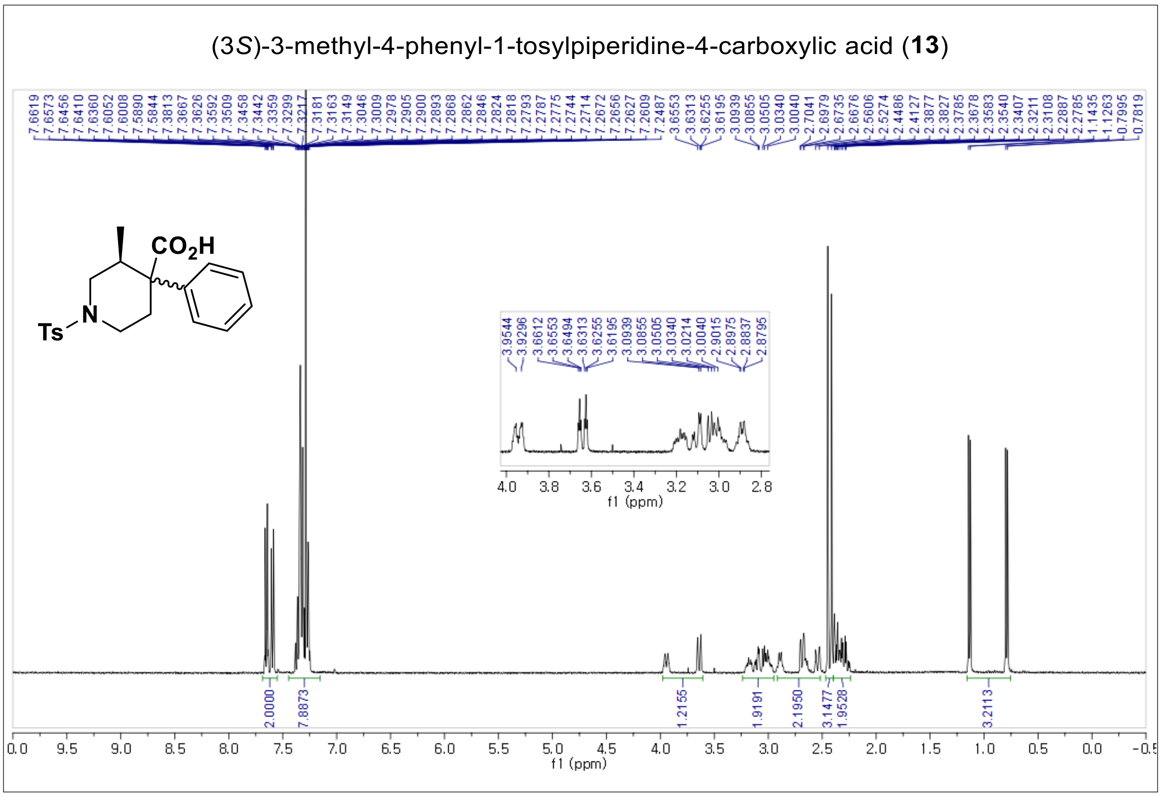

▼ ^{13}C-NMR (100MHz, $CDCl_3$) spectrum

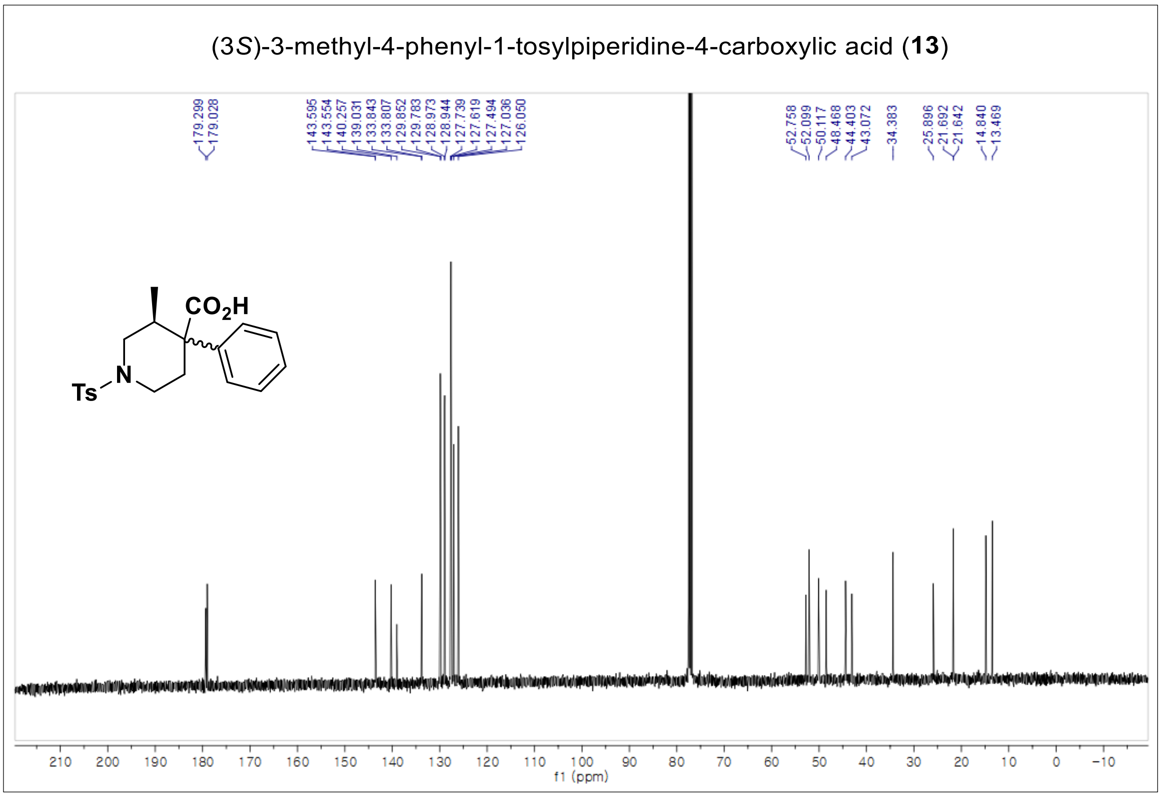

▼ High-Resolution Mass spectrum

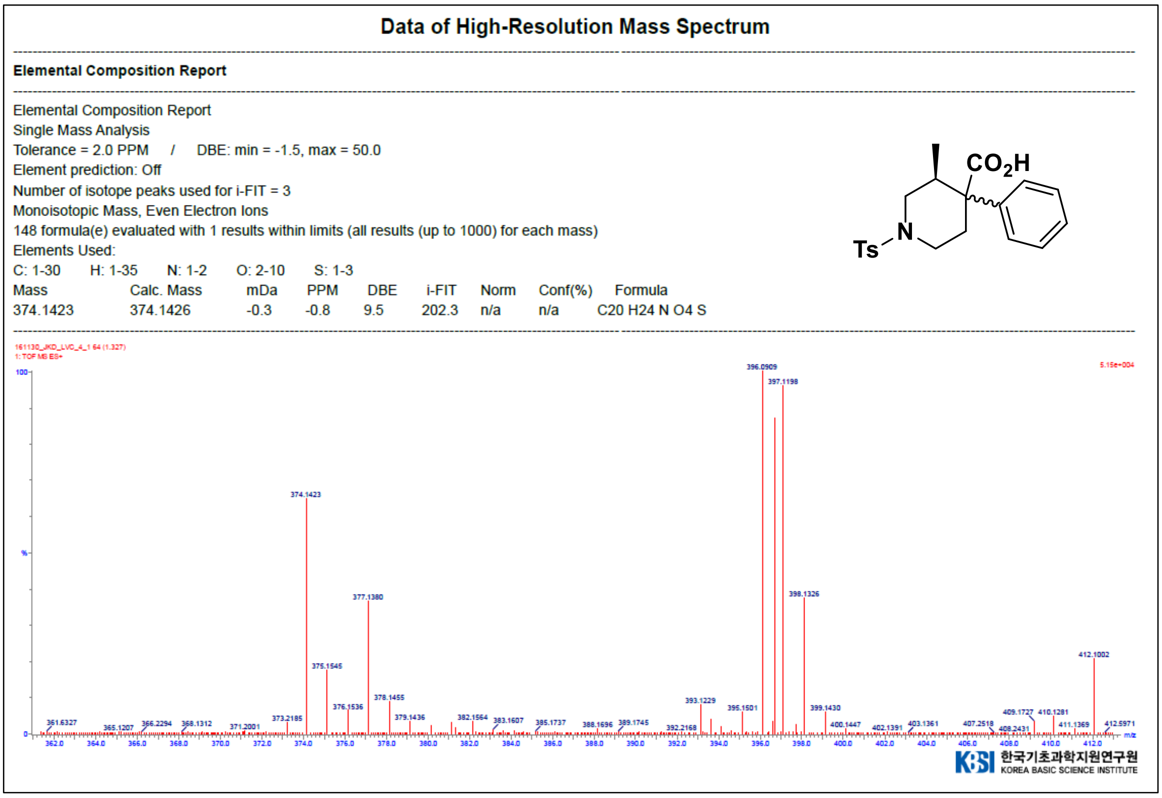

(3*S*,4*R*)-Benzyl-3-methyl-4-phenyl-1-tosylpiperidine-4-carboxylate (5)

▼ ^{1}H-NMR (400MHz, $CDCl_3$) spectrum

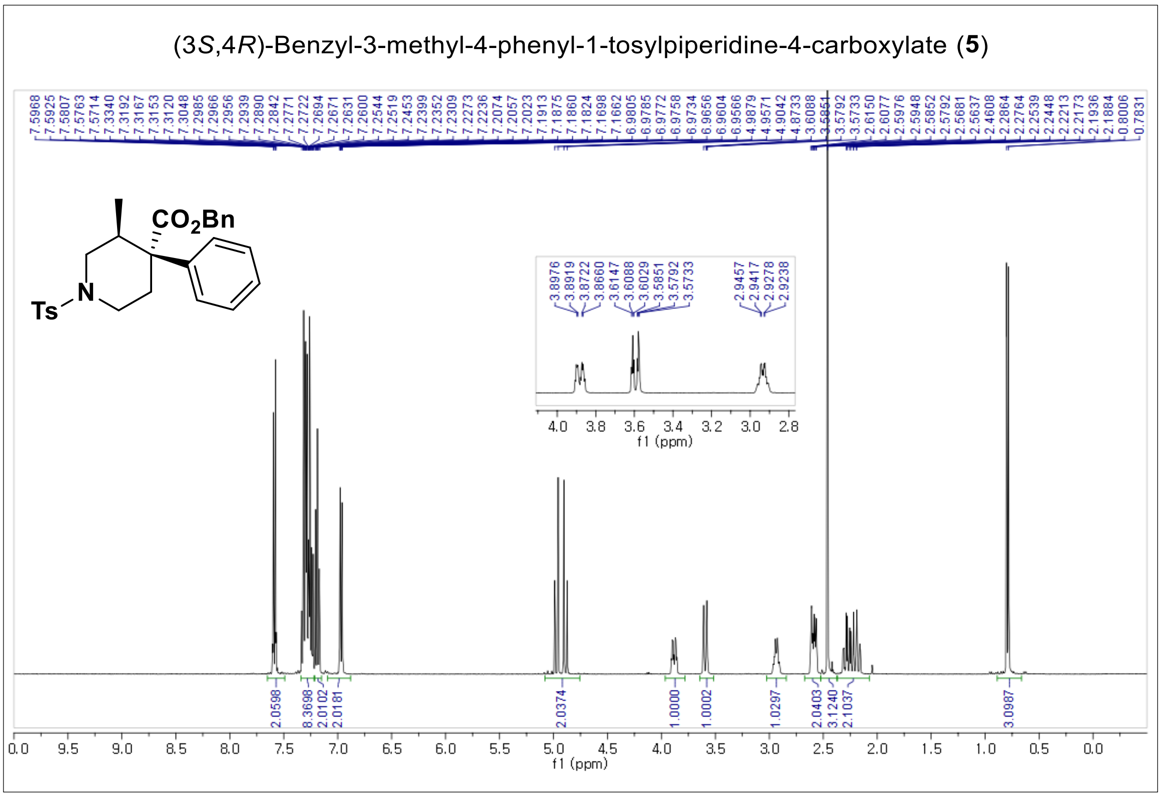

▼ ^{13}C-NMR (100MHz, $CDCl_3$) spectrum

(3*S*,4*R*)-Benzyl-3-methyl-4-phenyl-1-tosylpiperidine-4-carboxylate (**5**)

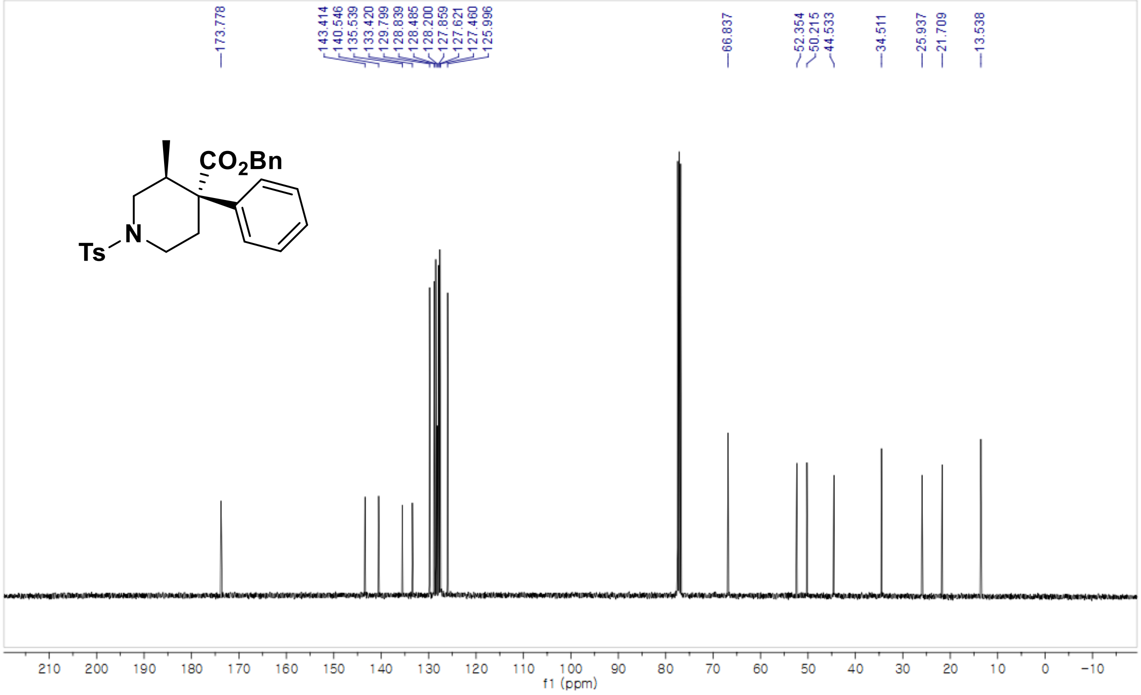

▼ High-Resolution Mass spectrum

Data of High-Resolution Mass Spectrum

Elemental Composition Report

Elemental Composition Report
Single Mass Analysis
Tolerance = 2.0 PPM / DBE: min = -1.5, max = 50.0
Element prediction: Off
Number of isotope peaks used for i-FIT = 3
Monoisotopic Mass, Even Electron Ions
151 formula(e) evaluated with 1 results within limits (all results (up to 1000) for each mass)
Elements Used:
C: 1-30 H: 1-35 N: 1-2 O: 2-10 S: 1-3

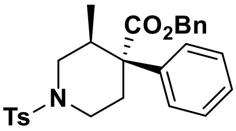

Mass	Calc. Mass	mDa	PPM	DBE	i-FIT	Norm	Conf(%)	Formula
464.1894	464.1896	-0.2	-0.4	13.5	153.6	n/a	n/a	C27 H30 N O4 S

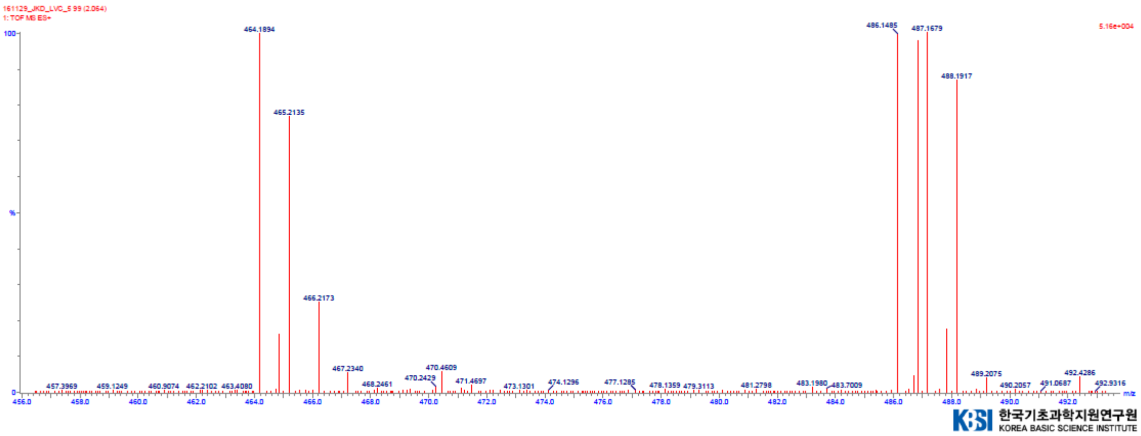

한국기초과학지원연구원
KOREA BASIC SCIENCE INSTITUTE

▼ HPLC (dr = 99.4 : 0.6)

===

Acq. Operator : Seq. Line : 3

Acq. Instrument : HS-LC9 Location : 62

Injection Date : 3/9/2016 1:21:22 PM Inj : 1

Inj Volume : 10.000 µl

Acq. Method : C:\Chem32\1\Data\Levovabastine\Levo_AJS 2016-03-09 12-04-51\Levocabastine(Step4, Step5).M

Last changed : 3/9/2016 12:04:51 PM

Analysis Method : C:\Chem32\1\Data\Everolimus-JJ\Everolimus 2016-11-16 12-16-24\Everolimus JJ.M (Sequence Method)

Last changed : 12/1/2016 1:08:32 PM (modified after loading)

Additional Info : Peak(s) manually integrated

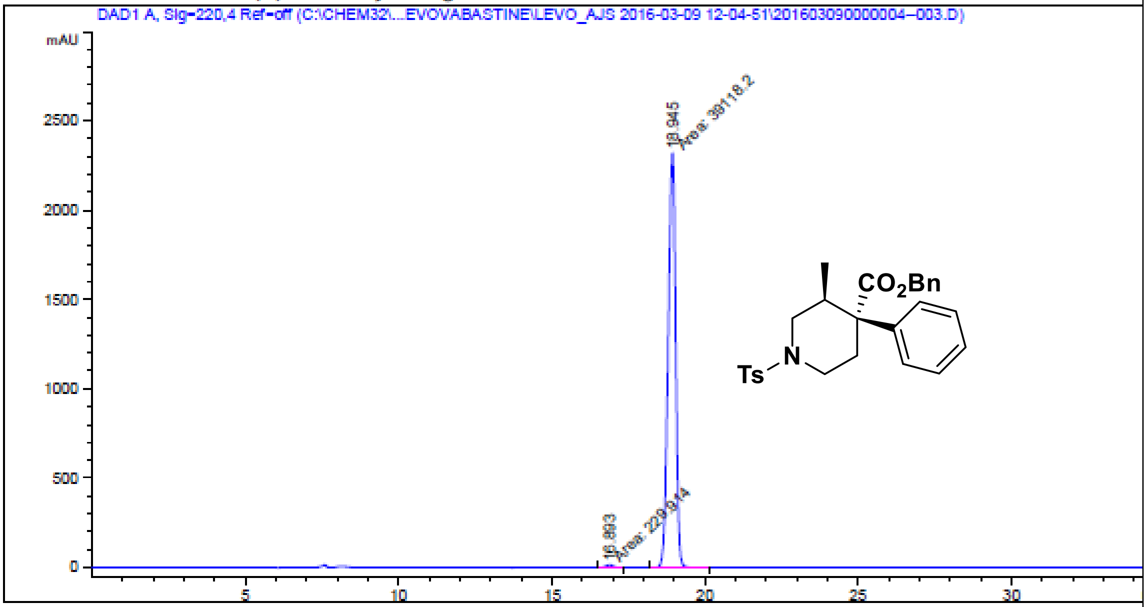

===

Area Percent Report

===

Sorted By : Signal

Calib. Data Modified : 11/17/2016 9:39:53 AM

Multiplier : 1.0000

Dilution : 1.0000

Do not use Multiplier & Dilution Factor with ISTDs

Signal 1: DAD1 A, Sig=220,4 Ref=off

Peak #	RetTime [min]	Type	Width [min]	Area [mAU*s]	Area %	Name
1	16.893	MM	0.2664	229.91402	0.5843	?
2	18.945	MM	0.2813	3.91182e4	99.4157	?

Totals : 3.93481e4

(3*S*,4*R*)-Benzyl-3-methyl-4-phenylpiperidine-4-carboxylate (6)

▼ ^{1}H-NMR (400MHz, CD_3OD) spectrum

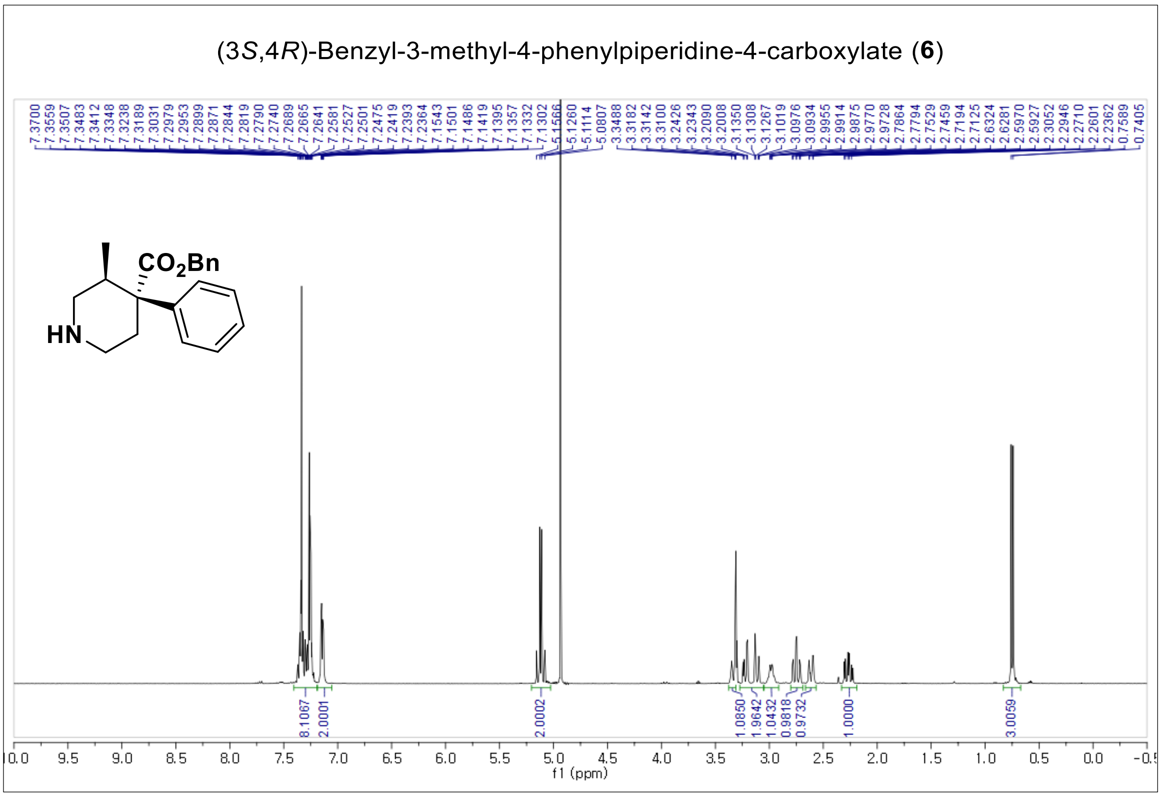

▼ ^{13}C-NMR (100MHz, CD_3OD) spectrum

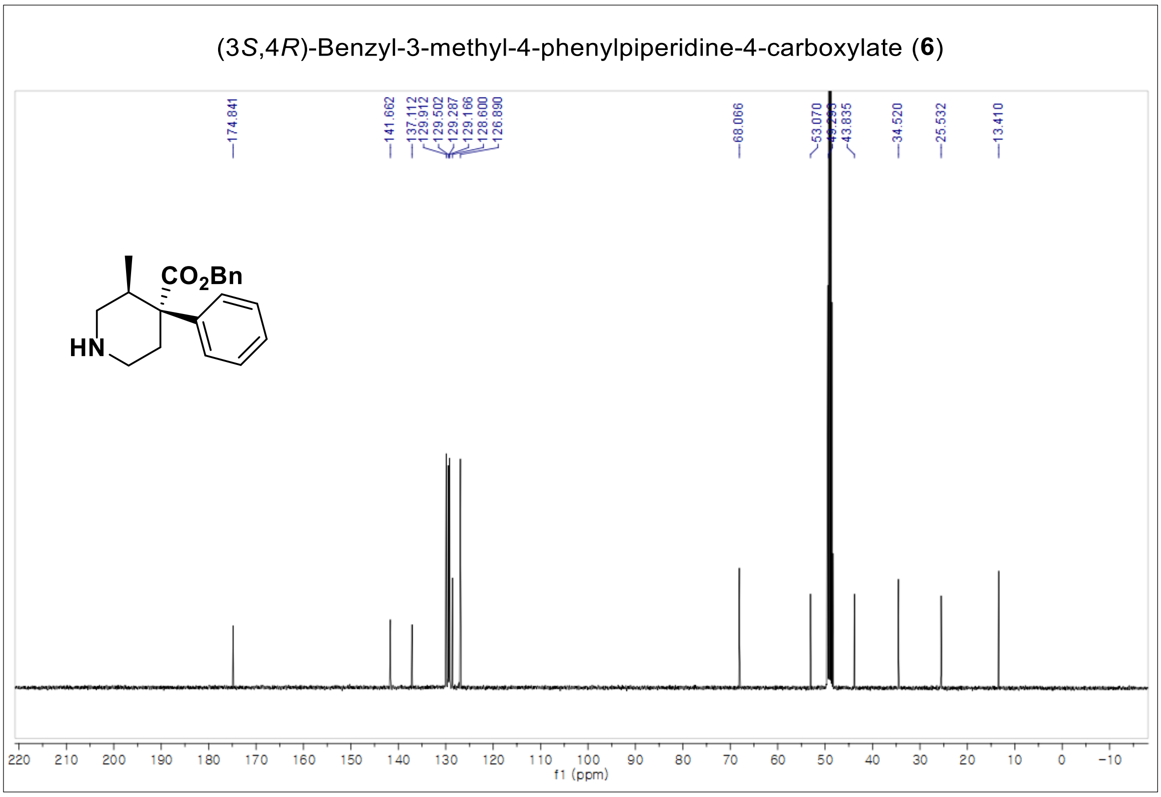

▼ High-Resolution Mass spectrum

Data of High-Resolution Mass Spectrum

Elemental Composition Report

Elemental Composition Report
Single Mass Analysis
Tolerance = 2.0 PPM / DBE: min = -1.5, max = 50.0
Element prediction: Off
Number of isotope peaks used for i-FIT = 3
Monoisotopic Mass, Even Electron Ions
51 formula(e) evaluated with 1 results within limits (all results (up to 1000) for each mass)
Elements Used:
C: 1-30 H: 1-35 N: 1-2 O: 2-10

Mass	Calc. Mass	mDa	PPM	DBE	i-FIT	Norm	Conf(%)	Formula
310.1811	310.1807	0.4	1.3	9.5	234.0	n/a	n/a	C20 H24 N O2

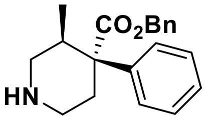

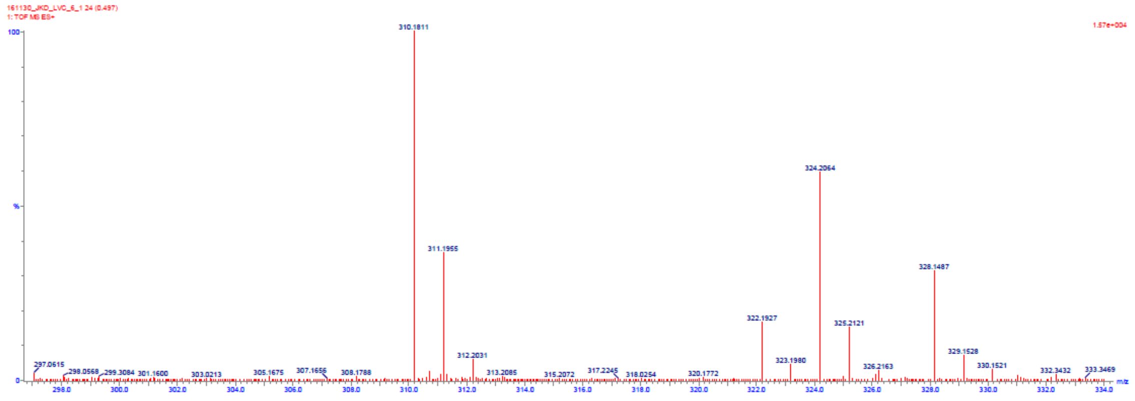

KBSI 한국기초과학지원연구원 KOREA BASIC SCIENCE INSTITUTE

(3*S*,4*R*)-benzyl-1-((1*S*,4*R*)-4-cyano-4-(4-fluorophenyl)cyclohexyl)-3-methyl-4-phenylpiperidine-4-carboxylate (8)

▼ ^{1}H-NMR (400MHz, $CDCl_3$) spectrum

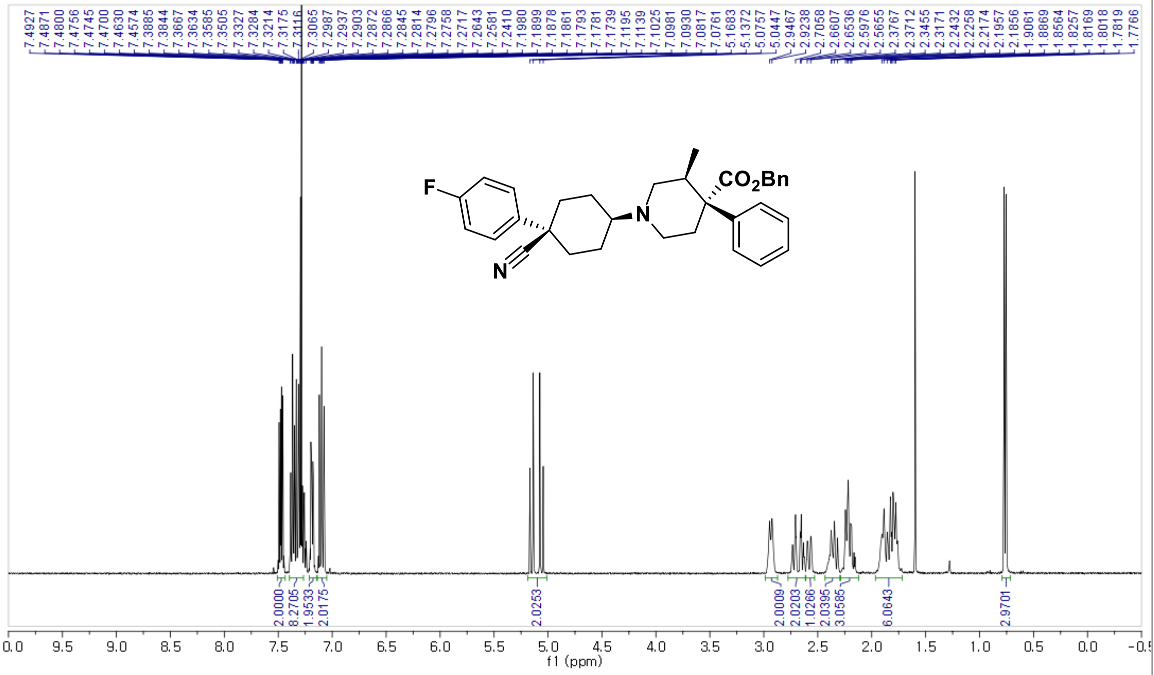

▼ ^{13}C-NMR (100MHz, $CDCl_3$) spectrum

(3*S*,4*R*)-benzyl-1-((1*S*,4*R*)-4-cyano-4-(4-fluorophenyl)cyclohexyl)-3-methyl-4-phenylpiperidine-4-carboxylate (**8**)

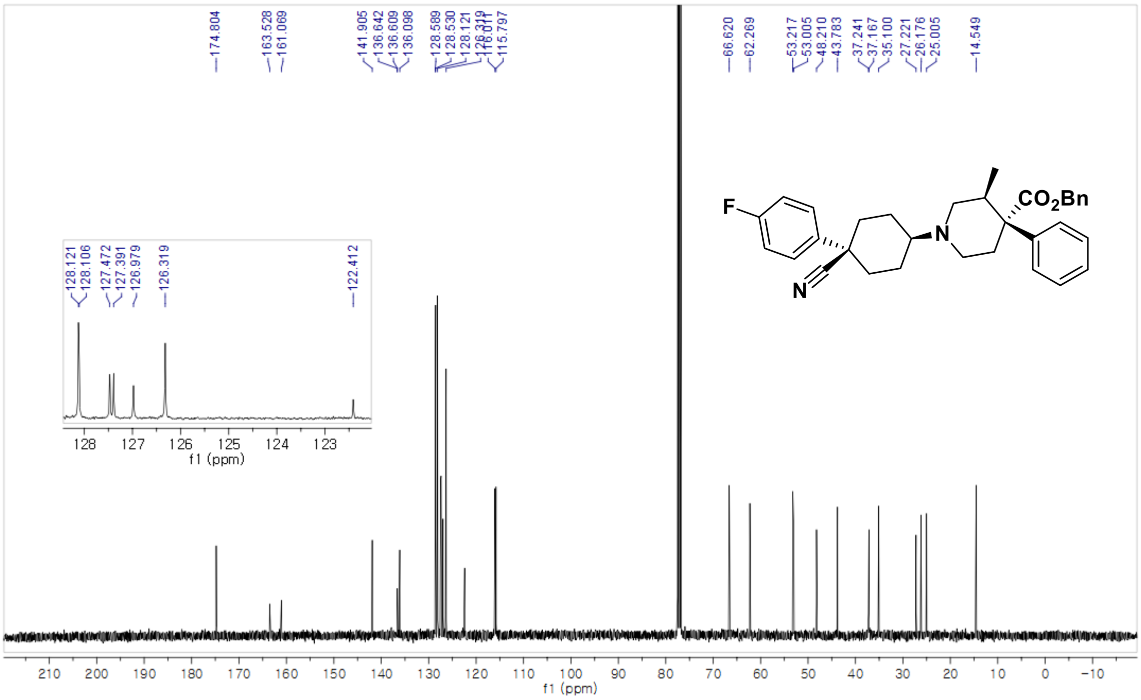

▼ High-Resolution Mass spectrum

Data of High-Resolution Mass Spectrum

Elemental Composition Report

Elemental Composition Report
Single Mass Analysis
Tolerance = 2.0 PPM / DBE: min = -1.5, max = 50.0
Element prediction: Off
Number of isotope peaks used for i-FIT = 3
Monoisotopic Mass, Even Electron Ions
118 formula(e) evaluated with 1 results within limits (all results (up to 1000) for each mass)
Elements Used:
C: 1-35 H: 1-40 N: 1-2 O: 2-10 F: 1-2

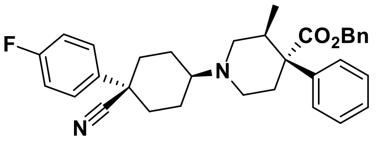

Mass	Calc. Mass	mDa	PPM	DBE	i-FIT	Norm	Conf(%)	Formula
511.2762	511.2761	0.1	0.2	16.5	178.5	n/a	n/a	C33 H36 N2 O2 F

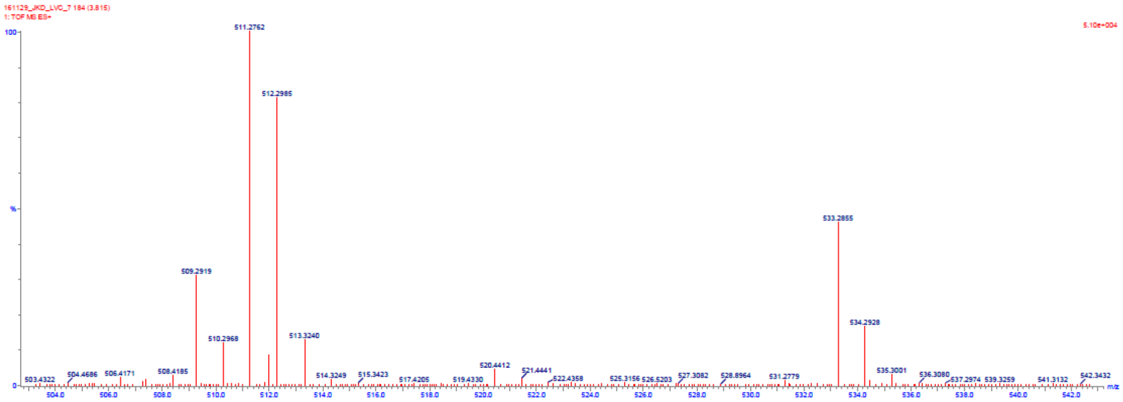

한국기초과학지원연구원
KOREA BASIC SCIENCE INSTITUTE

▼ HPLC (Purity : 99.5%)

===

[illegible]perator : LJM   Seq. Line : 4
Acq. Instrument : S4_HPLC   Location : Vial 45
Injection Date : 4/5/2016 6:36:30 PM   Inj : 1
Inj Volume : 2 µl
Different Inj Volume from Sequence !   Actual Inj Volume : 1 µl
Acq. Method : C:\Chem32\1\DATA\LEVOCABASTINE FINAL\LEVOCABASTINE FINAL 2016-04-05 17-00-22\LEVOCABASTINE_RA.M
Last changed : 3/16/2016 1:57:35 PM by LJM
Analysis Method : C:\CHEM32\1\METHODS\TESTOSTERONE.M
Last changed : 12/1/2016 12:35:35 PM by ndh
(modified after loading)

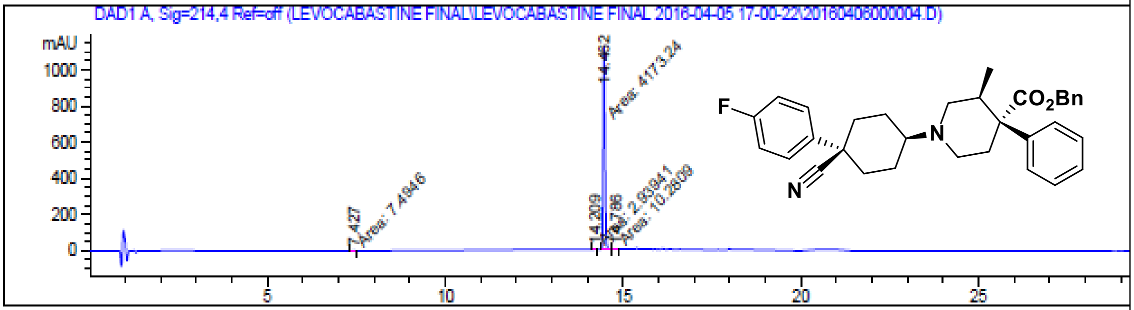

===

Area Percent Report

===

Sorted By : Signal
Multiplier : 1.0000
Dilution : 1.0000
Use Multiplier & Dilution Factor with ISTDs

Signal 1: DAD1 A, Sig=214,4 Ref=off

Peak #	RetTime [min]	Type	Width [min]	Area [mAU*s]	Height [mAU]	Area %
1	7.427	MM	0.1430	7.49460	8.73565e-1	0.1787
2	14.209	MM	0.0685	2.93941	7.14928e-1	0.0701
3	14.462	MF	0.0612	4173.23926	1137.14673	99.5061
4	14.786	FM	0.1272	10.28088	1.34661	0.2451

Totals : 4193.95415 1140.08183

===

*** End of Report ***

▼ HPLC (Geometric isomer < 0.1%)

===

[illegible]perator : LJM   Seq. Line : 4
Acq. Instrument : S4_HPLC   Location : Vial 45
Injection Date : 4/5/2016 6:36:30 PM   Inj : 1
                                                         Inj Volume : 2 µl
Different Inj Volume from Sequence !   Actual Inj Volume : 1 µl
Acq. Method : C:\Chem32\1\DATA\LEVOCABASTINE FINAL\LEVOCABASTINE FINAL 2016-04-05 17-00-22\LEVOCABASTINE_RA.M
Last changed : 3/16/2016 1:57:35 PM by LJM
Analysis Method : C:\CHEM32\1\METHODS\TESTOSTERONE.M
Last changed : 12/1/2016 12:35:35 PM by ndh
(modified after loading)

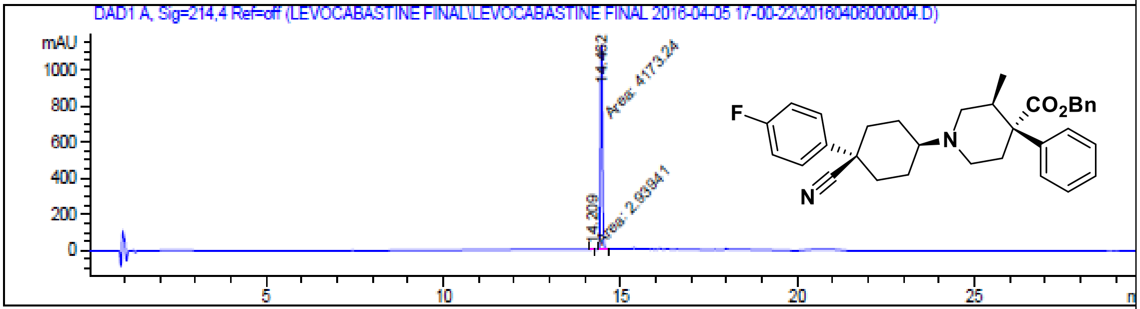

===

Area Percent Report

===

Sorted By : Signal
Multiplier : 1.0000
Dilution : 1.0000
Use Multiplier & Dilution Factor with ISTDs

Signal 1: DAD1 A, Sig=214,4 Ref=off

Peak #	RetTime [min]	Type	Width [min]	Area [mAU*s]	Height [mAU]	Area %
1	14.209	MM	0.0685	2.93941	7.14928e-1	0.0704
2	14.462	MF	0.0612	4173.23926	1137.14673	99.9296

Totals : 4176.17867 1137.86166

===

*** End of Report ***

(3*S*,4*R*)-1-(*cis*-4-cyano-4-(4-fluorophenyl)cyclohexyl)-3-methyl-4-phenyl-4-piperidinecarboxylic acid (1, levocabastine hydrochloride)

▼ ^{1}H-NMR (400MHz, CD_3OD) spectrum

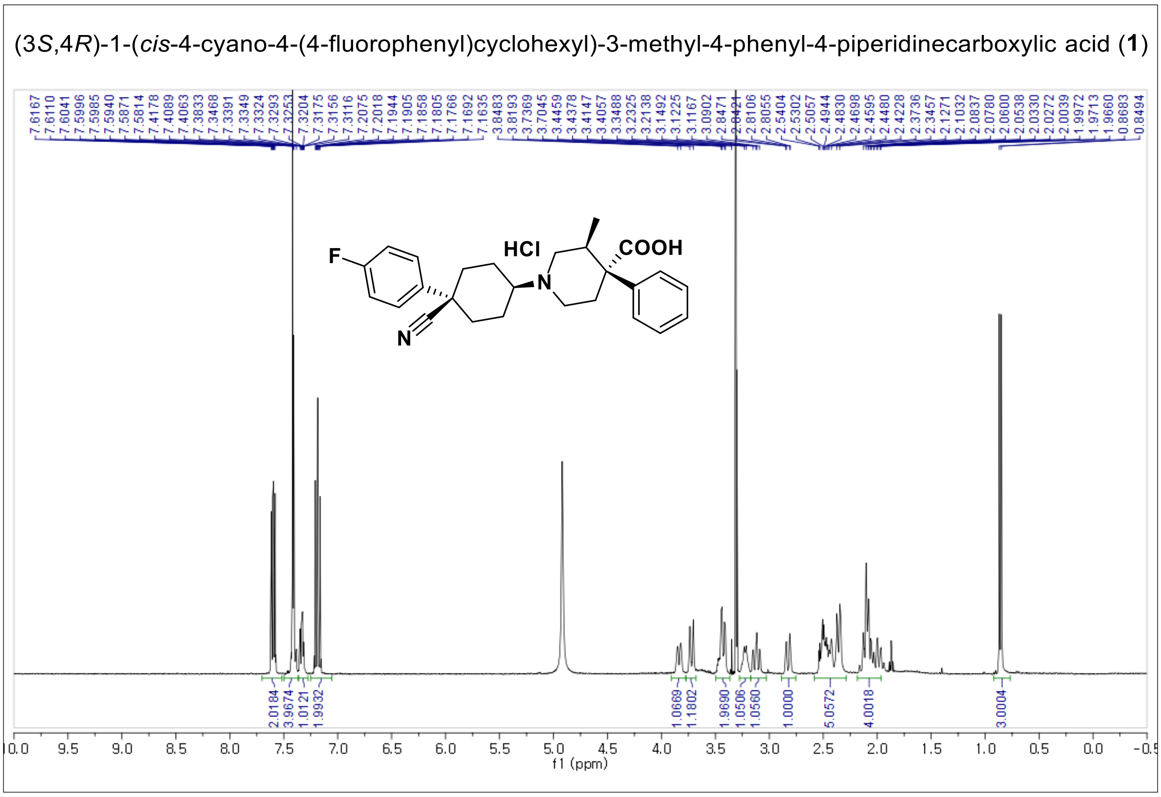

▼ ^{13}C-NMR (100MHz, CD_3OD) spectrum

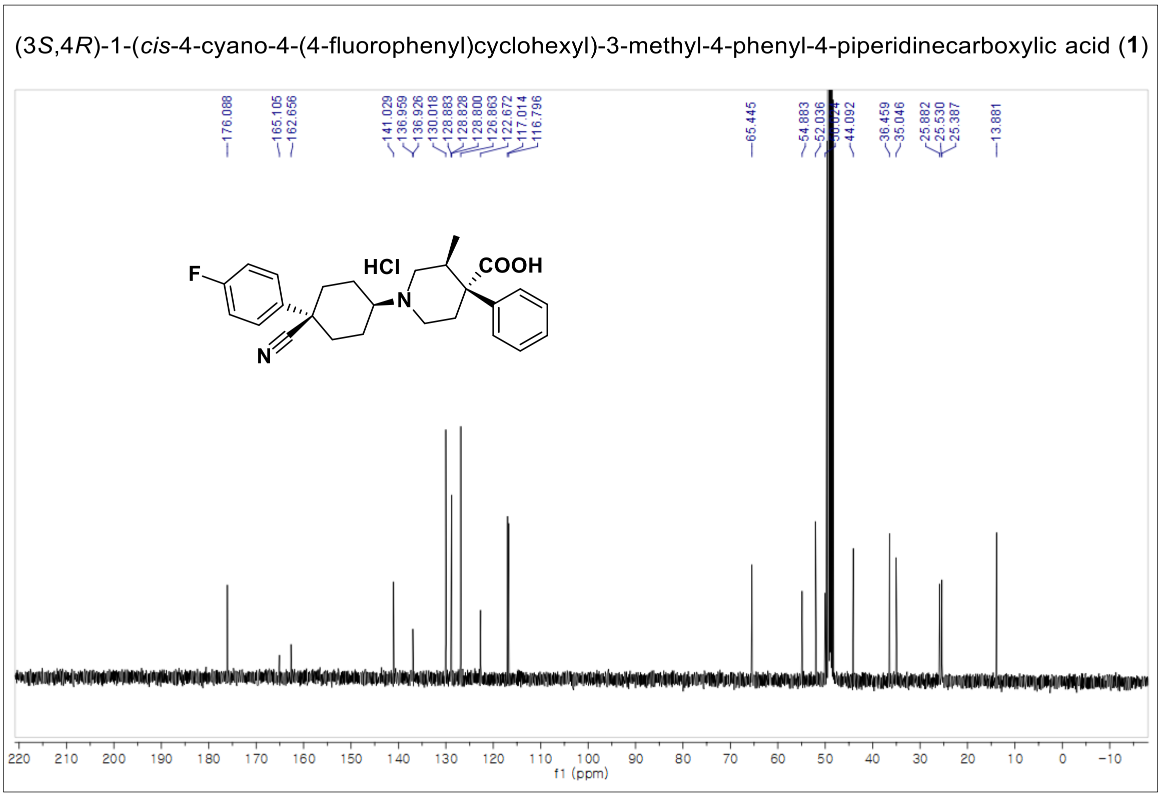

▼ High-Resolution Mass spectrum

Data of High-Resolution Mass Spectrum

Elemental Composition Report

Elemental Composition Report
Single Mass Analysis
Tolerance = 2.0 PPM / DBE: min = -1.5, max = 50.0
Element prediction: Off
Number of isotope peaks used for i-FIT = 3
Monoisotopic Mass, Even Electron Ions
119 formula(e) evaluated with 1 results within limits (all results (up to 1000) for each mass)
Elements Used:
C: 1-35 H: 1-40 N: 1-2 O: 2-10 F: 1-2

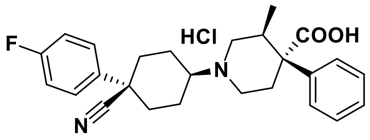

Mass	Calc. Mass	mDa	PPM	DBE	i-FIT	Norm	Conf(%)	Formula
421.2292	421.2291	0.1	0.2	12.5	207.5	n/a	n/a	C26 H30 N2 O2 F

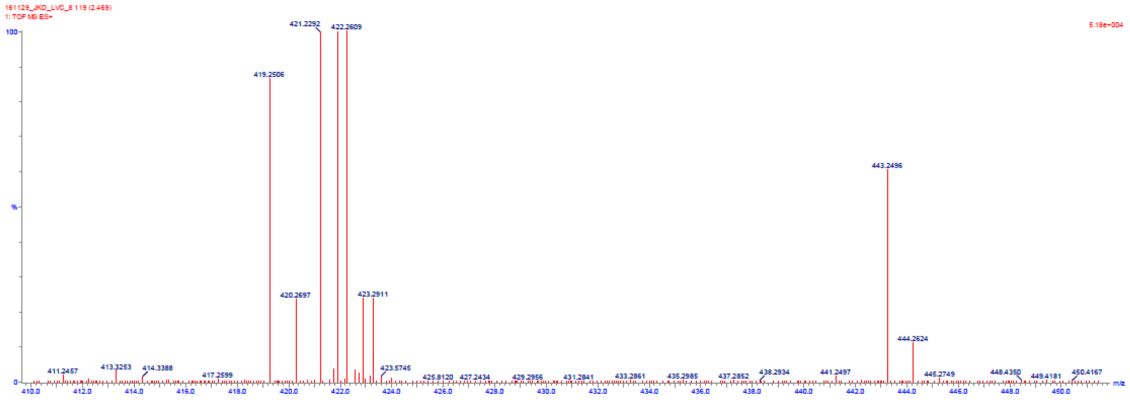

한국기초과학지원연구원
KOREA BASIC SCIENCE INSTITUTE

▼ HPLC (Purity = 99.7%)

===

[illegible]perator : LJM Seq. Line : 2
Acq. Instrument : S4_HPLC Location : Vial 56
Injection Date : 5/26/2016 8:11:53 PM Inj : 1
Inj Volume : 2 µl
Different Inj Volume from Sequence ! Actual Inj Volume : 1.5 µl
Acq. Method : C:\Chem32\1\DATA\LEVOCABASTINE FINAL\LEVOCABASTINE FINAL 2016-05-26 19-39-34\LEVOCABASTINE_RA.M
Last changed : 4/12/2016 11:50:13 AM by LJM
Analysis Method : C:\CHEM32\1\METHODS\TESTOSTERONE.M
Last changed : 12/1/2016 12:35:35 PM by ndh
(modified after loading)

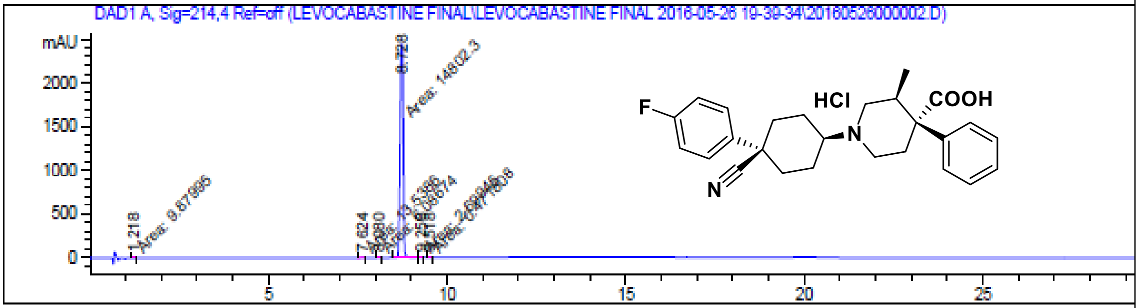

===

Area Percent Report

===

Sorted By : Signal
Multiplier : 1.0000
Dilution : 1.0000
Use Multiplier & Dilution Factor with ISTDs

Signal 1: DAD1 A, Sig=214,4 Ref=off

Peak #	RetTime [min]	Type	Width [min]	Area [mAU*s]	Height [mAU]	Area %
1	1.218	MM	0.0754	9.87995	2.18448	0.0666
2	7.624	MM	0.0844	13.53859	2.67203	0.0913
3	8.080	MM	0.0749	6.08674	1.35374	0.0410
4	8.728	MF	0.1012	1.48023e4	2437.61938	99.7797
5	9.256	FM	0.0922	2.69945	4.87852e-1	0.0182
6	9.518	MM	0.0697	4.71808e-1	1.12804e-1	3.180e-3

Totals : 1.48350e4 2444.43029

===

*** End of Report ***

▼ HPLC (Geometric isomer of levocabastine hydrochloride < 0.1%)

```
=====================================================================
[illegible]perator    : LJM                                  Seq. Line :    2
Acq. Instrument : S4_HPLC                              Location : Vial 56
Injection Date  : 5/26/2016 8:11:53 PM                       Inj :    1
                                                    Inj Volume : 2 µl
Different Inj Volume from Sequence !      Actual Inj Volume : 1.5 µl
Acq. Method     : C:\Chem32\1\DATA\LEVOCABASTINE FINAL\LEVOCABASTINE FINAL 2016-05-26 19-39-34\
                  LEVOCABASTINE_RA.M
Last changed    : 4/12/2016 11:50:13 AM by LJM
Analysis Method : C:\CHEM32\1\METHODS\TESTOSTERONE.M
Last changed    : 12/1/2016 12:35:35 PM by ndh
                  (modified after loading)
```

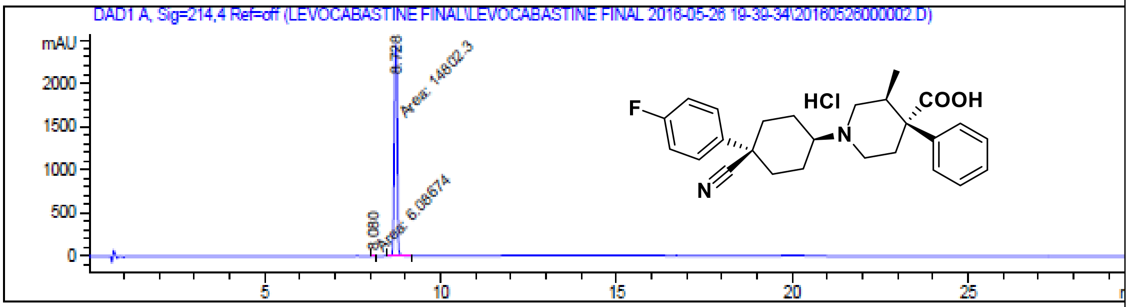


```
=====================================================================
                        Area Percent Report
=====================================================================

Sorted By             :      Signal
Multiplier            :      1.0000
Dilution              :      1.0000
Use Multiplier & Dilution Factor with ISTDs


Signal 1: DAD1 A, Sig=214,4 Ref=off

Peak RetTime Type  Width     Area      Height     Area
  #   [min]        [min]   [mAU*s]     [mAU]        %
----|-------|----|-------|----------|----------|--------|
   1   8.080 MM    0.0749    6.08674    1.35374   0.0411
   2   8.728 MF    0.1012 1.48023e4  2437.61938  99.9589

Totals :                   1.48084e4  2438.97313


=====================================================================
                          *** End of Report ***
```
